# Four newly synthesized enones induce mitochondrial-mediated apoptosis and G2/M cell cycle arrest in colorectal and cervical cancer cells†

**DOI:** 10.1039/d4ra06529h

**Published:** 2024-10-25

**Authors:** Marija Bulić, Ivana Nikolić, Marina Mitrović, Jovana Muškinja, Tamara Todorović, Marija Anđelković

**Affiliations:** a University Clinical Centre of Serbia, Centre of Medical Biochemistry Pasterova 2 Belgrade 11000 Serbia m.bulic81@yahoo.com; b Department of Biochemistry, University of Kragujevac, Faculty of Medical Sciences Svetozara Markovića 69 Kragujevac 34000 Serbia marijabcd@gmail.com angelkg2009@gmail.com mitrovicmarina34@gmail.com +381 65 84 777 68; c Centre for Research on Harmful Effects of Biological and Chemical Hazards Kragujevac Serbia; d Department of Science, University of Kragujevac, Institute for Information Technologies Jovana Cvijica bb Kragujevac 34000 Serbia jovana.muskinja@gmail.com tamaratasha34@gmail.com; e Center for Molecular Medicine and Stem Cell Research, Faculty of Medical Sciences, University of Kragujevac Svetozara Markovica 69 Kragujevac 34000 Serbia

## Abstract

Over the last few decades, we have gained insight into how researchers attempted to modify some natural molecules to be utilized as prospective agents for cancer treatment. Many scientists synthesized new natural compounds by incorporating specific functional groups and metals that improved their antitumor activity while reducing undesirable side effects. In this investigation, we synthesized four novel structurally modified enones that differ in the functional groups attached to the carbonyl group of the enone system (methyl – E1; isopropyl – E2; isobutyl – E3; and cyclopropyl – E4) and explored their anticancer potential against human carcinoma of the colon HCT-116, the cervical HeLa, and normal lung cells MRC-5. From the findings, all the newly synthesized enones exhibited potent cytotoxic activity against the cancer cells while normal cells remained unharmed, with varying potencies among the various enones. We employed the MTT assay to assess enones's (E1–E4) cytotoxic effects, IC50 values and selectivity index in tumor cells. Furthermore, the newly synthesized enones induced cell death in cancer cells through apoptosis by promoting changes in cellular morphology, activating apoptotic regulators Bax and caspase 3, and inhibiting Bcl-2. The enones induced changes in the mitochondrial membrane potential, a release of cytochrome c, and a cell cycle arrest at the G2/M phase, thus inhibiting the growth of cancer cells. In conclusion, we demonstrated the anticancer potential of newly synthesized enones as promising candidates for future cancer treatments, especially for colon cancer, due to their selective cytotoxicity against these cancer cells. Further, *in vivo* studies are warranted to explore their full therapeutic potential.

## Abstract 2

Mechanism of action for newly synthesized enones.

## Graphical abstract

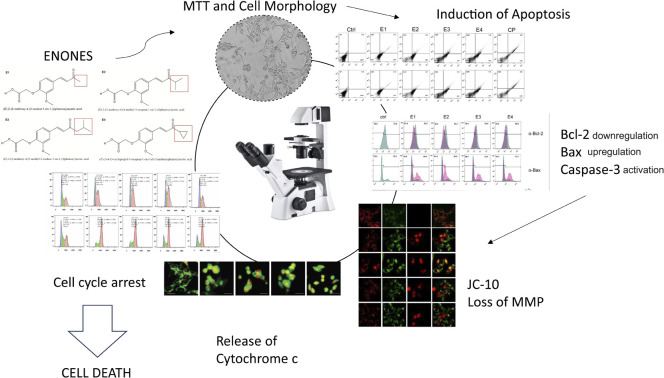

## 1 Introduction

**Fig. 1:**
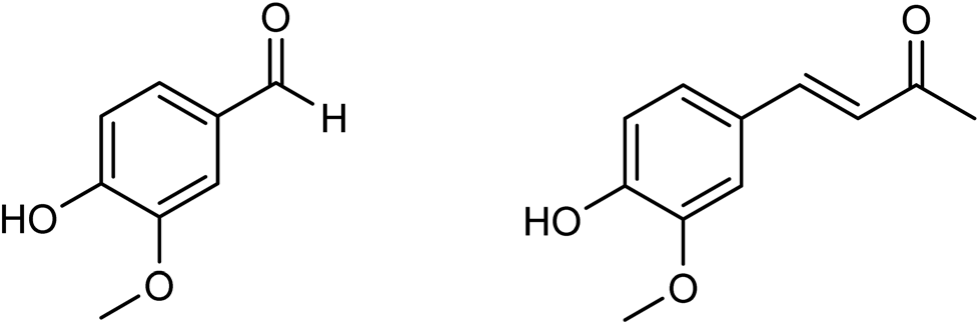
Structures of vanillin and dehydrozingerone.

In recent years, there has been a push toward searching for the golden standard for treating numerous different forms of cancer. There have, however, been some great advances in this area. So far significant results have been obtained regarding the anticancer efficacy of modified natural molecules or newly synthesized unique compounds.^1,2^ This modification can be achieved through the *de novo* synthesis of nature-inspired molecules or natural compounds that were chemically modified, for example by introducing specific functional groups or metals, thereby increasing their anticancer effects as well as general biological activity.^2^ In addition to tumor growth inhibition, these new compounds showed little toxicity against normal cells and did not cause the typical side effects experienced with cytostatic therapies. The purpose of this study is to investigate anticancer effects of four newly synthesized enones. These new potential therapeutic molecules were developed using a foundation of natural chemicals, including vanillin, 4-(4-hydroxy-3-methoxyphenyl)-3-buten-2-one, and dehydrozingerone. Chemical structure of vanillin and dehydrozingerone is presented in Fig. 1. For dehydrozingerone (DZG) and its analogues, several research studies offered data for anticancer activity in different types of cancer cells.^3–5^

**Fig. 2:**
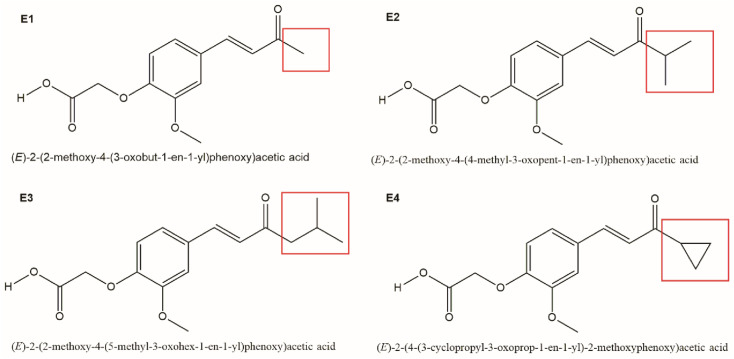
Molecular structural drawings of newly synthesized and further studied enones (E1–E4) with various substituents indicated by red squares.

In connection with our ongoing research project devoted to the synthesis of novel compounds based on natural products and their modifications thereafter, four new enones having different functional groups at carbonyl moieties (C-3; C-α) were synthesized. In detail, we substituted the carbonyl group of the enone system with methyl (E1), isopropyl (E2), isobutyl (E3), and cyclopropyl (E4) groups based on the transformation route plan shown in Fig. 2 (the red squares were substitution sites). Based on this reactivity, and its thorough documentation in literature, it seemed rational to us that the introduction of new functional groups would generate efficient multitargeting therapeutic agents. According to this idea, four new O-acetyl derivatives: enone 1 (E1), enone 2 (E2), enone 3 (E3) and enone E4 were synthesized. The purpose of this study was to evaluate the potential antitumor effects of these new chemically synthesized compounds containing the modified chemical structures of the groups attached to the carbonyl group and to establish whether they possessed more efficient anticancer activity than commercially available cytostatic cisplatin.

In this study, we explored the potential anticancer activity of four novel structurally modified enones E1–E4 against human colon cancer HCT-116, cervical cancer HeLa, and normal lung cells MRC-5. We also investigated the underlying mechanism of their cytotoxicity with a view toward their potential application as antitumor drugs. Additionally, the study aimed to confirm the anticancer activity of enones compared to commercially used cytostatic (CP). A key focus of the research was to assess the selectivity and non-toxicity of the newly synthesized enones toward healthy cells.

## 2 Experimental

### 2.1. Chemistry

**Scheme 1:**
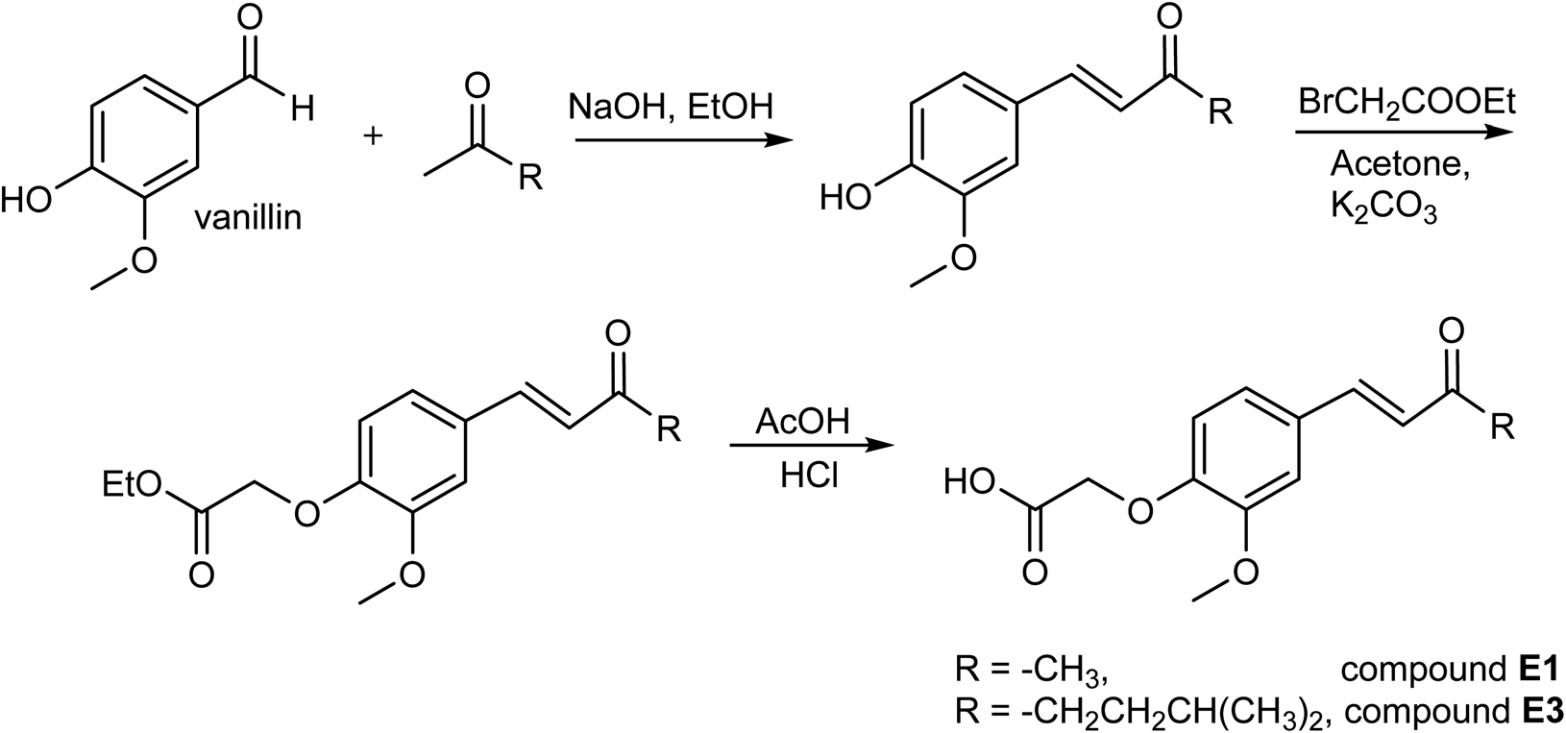
Synthesis of enones E1 and E3.

**Scheme 2:**
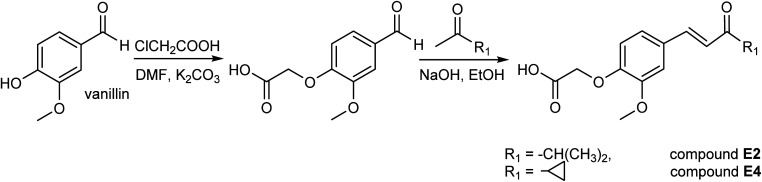
Synthesis of enones E2 and E4.

The four new enones (E1–E4) were obtained in very good yields, and their synthesis is shown in Scheme 1 and 2. The structures of the novel products were characterized by spectral data (^1^HNMR and ^13^CNMR).

All starting chemicals were commercially available and used as received, except the solvents were purified by distillation. IR spectra: PerkinElmer Spectrum One FT-IR spectrometer with a KBr disc, in cm^−1^; NMR spectra: Varian Gemini 200 MHz spectrometer (200 MHz for ^1^H and 50 MHz for ^13^C), using DMSO-d_6_ with 0.02% water and TMS as the internal standard. ^1^H and ^13^C NMR chemical shifts were reported in parts per million (ppm) and were referenced to the solvent peak. Multiplicities are represented by s (singlet), d (doublet), t (triplet), dd (doublet of doublets), and m (multiplet). Coupling constants (*J*) are in Hertz (Hz). The melting point of products was determined by using the MelTemp1000 apparatus.

### 2.2. General procedures for the synthesis of enones E1 and E3

Enone compounds (E1 and E3) were obtained in the reaction shown in Scheme 1.

Starting from vanillin and various methyl ketones, some dehydrozingerone and its analogues were prepared, following our previously described literature procedures.^6–8^ In the further reaction, these phenol products (10 mmol) were dissolved in 40 ml acetone, 15 mmol (2.5 g) of ethyl bromoacetate and 50 mmol (7 g, anhydrous) of K_2_CO_3_ were added, and the mixture was stirred at reflux overnight. Acetone and excess ester were evaporated under reduced pressure and solid residue was dissolved in water and extracted with CH_2_Cl_2_ (3 × 50 ml). The organic layer was washed with water (2 × 50 ml) and brine (2 × 50 ml) and dried. The main part of the solvent was evaporated at reduced pressure and a crude concentrated solution was filtered through a short column of silica gel yielding solid esters.

The corresponding ester (5 mmol) was dissolved in 10 ml of acetic acid and 5 ml of 2 M HCl was added, and the reaction mixture was heated for 30 minutes at 95 °C. The resulting solution was cooled and poured into 100 g of crushed ice. After standing in freeze overnight, the solid separated was filtered and dried. The enones E1 and E3 were obtained as white solid compounds.

### 2.3. General procedures for the synthesis of enones E2 and E4

Enone compounds (E2 and E4) were obtained in the reaction shown in Scheme 2.

The vanillin (10 mmol, 1.52 g) was dissolved in 15 ml DMF, and chloroacetic acid (11 mmol, 1.1 g) and K_2_CO_3_ (20 mmol, 2.8 g) were added. The reaction mixture was heated overnight at 50 °C. The resulting solution was cooled and poured into 100 g of crushed ice. The reaction mixture was neutralized with 3 M HCl to get the powdery precipitate. After standing in freeze overnight, the solid separated was filtered and dried. The obtained alkylated vanillin was used for further reaction.

The obtained alkylated vanillin (1 mmol) and corresponding methyl ketone (20 mmol) were dissolved in 15 ml of ethanol, the mixture was stirred for 10 min, and 25 ml 2% NaOH was added slowly. The reaction mixture was stirred at room temperature overnight. The resulting solution was cooled and poured into water. After standing in freeze overnight, the solid separated was filtered and dried.

#### 2.3.1 (*E*)-2-(2-Methoxy-4-(3-oxobut-1-en-1-yl)phenoxy)acetic acid (E1)

Yield: 85.2%; m.p. 143–144 °C; IR (KBr): 3469, 2920, 1715, 1626, 1594, 1509, 1417, 1227, 1134, 1026, 972, 804 cm^−1^; ^1^HNMR (200 MHz, DMSO-d_6_): 2.31 (s, 3H, CH_3_CO), 3.83 (s, 3H, OCH_3_), 4.74 (s, 2H, OCH_2_), 6.74 (d, 1H, *J* = 16.4 Hz, CH), 6.89 (d, 1H, *J* = 8.4 Hz, Ar–H), 7.22 (dd, 1H, *J* = 8.4, 2.0 Hz, Ar–H), 7.35 (d, 1H, *J* = 1.6 Hz, Ar–H), 7.55 (d, 1H, *J* = 16.4 Hz, CH), 13.10 (br. s, 1H, COOH); ^13^CNMR (50 MHz, DMSO-d_6_): 27.3, 55.8, 65.1, 111.3, 113.1, 122.5, 125.6, 127.9, 143.2, 149.1, 149.4, 169.8, 197.7 (CO).

#### 2.3.2 (*E*)-2-(2-Methoxy-4-(4-methyl-3-oxopent-1-en-1-yl)phenoxy)acetic acid (E2)

Yield: 62.7%; m.p. 115–116 °C; IR (KBr): 3508, 2911, 1755, 1575, 1509, 1423, 1270, 1207, 1141, 1012, 977, 795 cm^−1^; ^1^HNMR (200 MHz, DMSO-d_6_): 1.07 (d, 6H, *J* = 6.8 Hz, 2CH_3_), 2.91–3.05 (*m*, 1H, C*H*(CH_3_)_2_), 3.83 (s, 3H, OCH_3_), 4.73 (s, 2H, OCH_2_), 6.86–6.98 (m, 1H, Ar–H), 6.94 (d, 1H, *J* = 16.0 Hz, CH), 7.23 (dd, 1H, *J* = 8.4, 1.8 Hz, Ar–H), 7.39 (d, 1H, *J* = 1.8 Hz, Ar–H), 7.54 (d, 1H, *J* = 16.0 Hz, CH), 13.09 (br. s, 1H, COOH); ^13^CNMR (50 MHz, DMSO-d_6_): 18.6, 38.2, 55.9, 65, 111.3, 113, 122.7, 123.1, 128, 141.9, 149.3, 149.4, 169.8, 202.9 (CO).

#### 2.3.3 (*E*)-2-(2-Methoxy-4-(5-methyl-3-oxohex-1-en-1-yl)phenoxy)acetic acid (E3)

Yield: 93.2%; m.p. 117–118 °C; IR (KBr): 3494, 2957, 1748, 1578, 1509, 1421, 1267, 1208, 1137, 1030, 819, 793 cm^−1^; ^1^HNMR (200 MHz, DMSO-d_6_): 0.91 (d, 6H, *J* = 6.6 Hz, 2CH_3_), 2.05–2.18 (m, 1H, C*H*(CH_3_)_2_), 2.53 (d, 2H, *J* = 7.0 Hz, C*H*_2_CH(CH_3_)_2_), 3.83 (s, 3H, OCH_3_), 4.73 (s, 2H, OCH_2_), 6.81 (d, 1H, *J* = 16.4 Hz, CH), 6.90 (s, 1H, Ar–H), 7.22 (dd, 1H, *J* = 8.4, 1.8 Hz, Ar–H), 7.36 (d, 1H, *J* = 1.8 Hz, Ar–H), 7.54 (d, 1H, *J* = 16.2 Hz, CH), 13.06 (br. s, 1H, COOH); ^13^CNMR (50 MHz, DMSO-d_6_): 22.6, 24.8, 48.9, 55.8, 65, 111.3, 113, 122.6, 125.1, 128, 142.2, 149.1, 149.4, 169.8, 199.5 (CO).

#### 2.3.4 (*E*)-2-(4-(3-Cyclopropyl-3-oxoprop-1-en-1-yl)-2-methoxyphenoxy)acetic acid (E4)

Yield: 90.8%; m.p. 89–90 °C; IR (KBr): 3416, 1722, 1633, 1510, 1426, 1393, 1224, 1144, 1077, 989, 787 cm^−1^; ^1^HNMR (200 MHz, DMSO-d_6_): 0.93 (d, 4H, *J* = 6.2 Hz, 2CH_2_), 2.36–2.45 (m, 1H, CH), 3.84 (s, 3H, OCH_3_), 4.74 (s, 2H, OCH_2_), 6.89 (d, 1H, *J* = 8.4 Hz, Ar–H), 6.98 (d, 1H, *J* = 16.0 Hz, CH), 7.24 (dd, 1H, *J* = 8.4, 1.6 Hz, Ar–H), 7.39 (d, 1H, *J* = 1.8 Hz, Ar–H), 7.62 (d, 1H, *J* = 16.2 Hz, CH), 13.07 (br. s, 1H, COOH); ^13^CNMR (50 MHz, DMSO-d_6_): 10.6, 19, 55.9, 65.1, 111.3, 113, 122.8, 124.8, 128, 142, 149.2, 149.4, 169.9, 199.1 (CO).

### 2.4. Cell lines, chemicals and reagents

In this study two cancer cell lines were used: human colon (HCT-116, ATCC-CCL-247) and human cervical (HeLa, ATCC-CCL-2) cancer cell line. For the purpose of evaluating the effect of compounds on normal cells, human fibroblast lung cell line (MRC-5) was used (ATCC® CCL-2™). The cells were maintained and cultivated at 37 °C and 5% CO_2_ in full growth DMEM medium (Dulbecco's Modified Eagle's medium, Sigma-Aldrich D5671). For the purpose of making full growth medium, DMEM was supplemented with 1% non-essential amino acid (Sigma-Aldrich, M7145), 100 U ml^−1^ penicillin, 100 μg ml^−1^ streptomycin (Roche, 11074440001) and 10% fetal bovine serum (FBS, Sigma-Aldrich, F7524).

Cisplatin was purchased from Sigma-Aldrich (St Louis, MO; CAS 15663-27-1). Newly enones were synthesized by Jovana Muškinja (University of Kragujevac, Department of Science, Institute for Information Technologies).

### 2.5. Cytotoxicity assessment by MTT assay

To examine and compare the antiproliferative activities of four enones, the cytotoxicity of the compounds was assessed by the 3-(4,5-dimethylthiazol-2-yl)-2,5-diphenyltetrazolium bromide (MTT) assay^9^ in, colon cancer cells (HCT-116), cervical cancer cell line (HeLa) and human fibroblast lung cell line (MRC-5) that was used as a healthy control cell line. After trypsinization, seeded (5 × 10^3^ cells per well in 96-well plates) cells were treated with different concentrations of the investigated enones (1, 3, 10, 30, and 100 μM) during 48 and 72 h period incubation period +37 °C and 5–6.5% CO_2_. After the incubation period, the medium was removed and MTT solution (5 mg ml^−1^) was added to the wells.

After a 3 hour incubation with the MTT solution, the resulting formazan crystals were dissolved in DMSO (67-68-5, Merck). Absorbance was subsequently measured at 595 nm using a microplate reader (Zenith 3100, Anthos Labtech Instruments). The percentage of cytotoxic cells was determined using the formula: cytotoxicity (%) = (1 − (exp. group (ABS))/(control group (ABS)) × 100). IC_50_ values were calculated using GraphPad Prism 10 software. The selectivity index (SI) was determined using the formula: SI=IC_50_ value for healthy cells/IC_50_ value for tumor cells.^9^

### 2.6. Analysis of cell morphology

Cancer and control cells were seeded and incubated for 48 and 72 hours with varying concentrations of enones (E1–E4). Cell analysis was performed following the method described by Luković *et al.*^9^ The cellular morphology was visualized after incubation using phase-contrast microscopy (100× magnification, Olympus IX50 inverted microscope). Morphological changes were subsequently analyzed using ImageJ software.

### 2.7. Assessment of apoptosis by annexin V-FITC/7-AAD staining

The type of cell death was assessed using flow cytometry using FITC Annexin V Apoptosis Detection Kit with 7-AAD (640922, Bio legend).^8^ Cells were seeded at a density of 1 × 10^5^ cells per well in 24-well plates and treated with IC_50_ concentrations of the enones for 48 and 72 h. After treatment, cells were harvested, washed with PBS (resuspended in 100 μl of ice-cold 1X binding buffer), stained (20 μl of 7-AAD and 10 μl of Annexin V-FITC), and incubated in the dark for 15 min at +4 °C. After incubation, 400 μl of binding buffer was added and the samples were analyzed using flow cytometer Cytomics FC500 (Beckman Coulter, USA). Data analysis was performed using FlowJo Software and presented with histogram and dot plots.

### 2.8. Flow cytometric analysis of apoptosis-related proteins

HCT-116 and HeLa cells were treated with IC_50_ concentrations of enones while untreated control, HCT-116 and HeLa cells were incubated in media alone for 48 hours. Cells were washed with PBS, fixed, permeabilized using Fixation and Permeabilization Kit (eBioscience), and stained separately for Bcl-2, Bax, and cleaved-caspase-3.^8,9^ Permeabilized cells were incubated with primary antibodies: mouse anti-human-Bcl-2 (DC21, sc-783, Santa Cruz Biotech. Inc.), mouse anti-human Bax (N20, sc-493, Santa Cruz Biotech. Inc.), and anti-cleaved caspase-3 (Cell signaling Technology 9661). Following antibody incubation, cells were washed and incubated with secondary Alexa 488-conjugated antibodies (ab185015, Abcam, United Kingdom) for 30 minutes. After an additional wash with PBS, flow cytometry analysis was performed.

Data were analyzed using FlowJo Software. The expression of Bcl-2 and Bax proteins was quantified as mean fluorescence intensity (MFI). The results were presented with bar graphs. Bax/Bcl-2 index was calculated using the formula Bcl-2 MFI/Bax MFI. The percentage of cells expressing active caspase-3 (MFI) was presented in histograms.

### 2.9. Cell cycle analysis

After treatment of HCT-116 and HeLa cells with enones, cells were harvested, washed with PBS, and fixed in 70% ice-cold ethanol at +4 °C overnight.^10^ The cells were then pelleted, resuspended in 1 ml PBS containing RNAse A (12091021, Sigma Aldrich), and incubated for 30 min at 37 °C. A staining solution containing propidium iodide (1 mg PI per ml PBS) (B7758, Biotrend) was added to cells, followed by 15 min incubation in the dark. Cells were analyzed by flow cytometer Cytomics FC500. DNA content was determined using Flowing Software and cell cycle distribution was presented by graph (histograms).

### 2.10. Determination of expression and localization of cytochrome c using immunofluorescence method

After 48 hours of treatment, immunofluorescence analysis of cytochrome c distribution and expression in treated cells was performed using glass coverslips.^10^ Following the treatment, cells were washed with PBS, fixed in 4% formaldehyde for 20 min, and permeabilized with 0.2% Tween-20. Blocking was performed using a buffer containing 0.1% Tween-20 and 10% FBS for 30 min. The cells were then incubated for 1 hour at room temperature with a monoclonal mouse anti-human cytochrome *c* antibody (1:100, G7421, Promega, USA,)

Subsequently, the cells were incubated for 30 minutes with a goat anti-mouse FITC (1:200, diluted in blocking buffer), washed, and images were acquired using an inverted fluorescence microscope (Olympus IX50). Image analysis was performed using ImageJ software.

### 2.11. Determination of mitochondrial membrane potential (Δ*Ψm*)– JC-10 analysis

Changes in the Δ*Ψm* caused by 48 hours of enones treatment, were assessed using JC-10 dye.^10^. (Enzo Life Sciences, Farmingdale, NY, USA).

In healthy cells, JC-10 dye accumulates in the mitochondria as red fluorescent aggregates. However, upon cell damage, changes in Δ*Ψm* occur and result in dislocation of JC-10 from the mitochondria to the cytosol and the monomerization of aggregates, shifting fluorescence from red to green. Cells were cultured in 24-well plate (3 × 10^4^ cells per well) overnight in the incubator (5% CO_2_, 37 °C). Treatment was conducted using IC_50_ concentrations of enones (E1–E4) for 48 hours. Following treatment, cells were washed with warm 1xPBS and incubated with JC-10 dye (2.5 μM in warm PBS) at 5% CO_2_, 37 °C for 20 minutes. Cells were then washed with 1xPBS and observed using an Olympus IX50 inverted fluorescence microscope. Images were captured and analyzed using ImageJ software. The ratio of fluorescence emissions at 525 and 590 nm was used for quantification analysis.

### 2.12. Statistical analysis

Experimental data were analyzed using the software SPSS version 20. The normality of data distribution was checked with the appropriate test depending on the sample size. Student's *t*-test was applied for normally distributed data, while the non-parametric Mann–Whitney test was used for non-normally distributed data. Results are presented as means ± standard error (SE). A statistically significant difference was found for (*p* < 0.05* and *p* < 0.01**).

## 3 Results

### 3.1. Newly synthesized enones exhibit cytotoxicity towards HCT-116 and HeLa cells

**Fig. 3:**
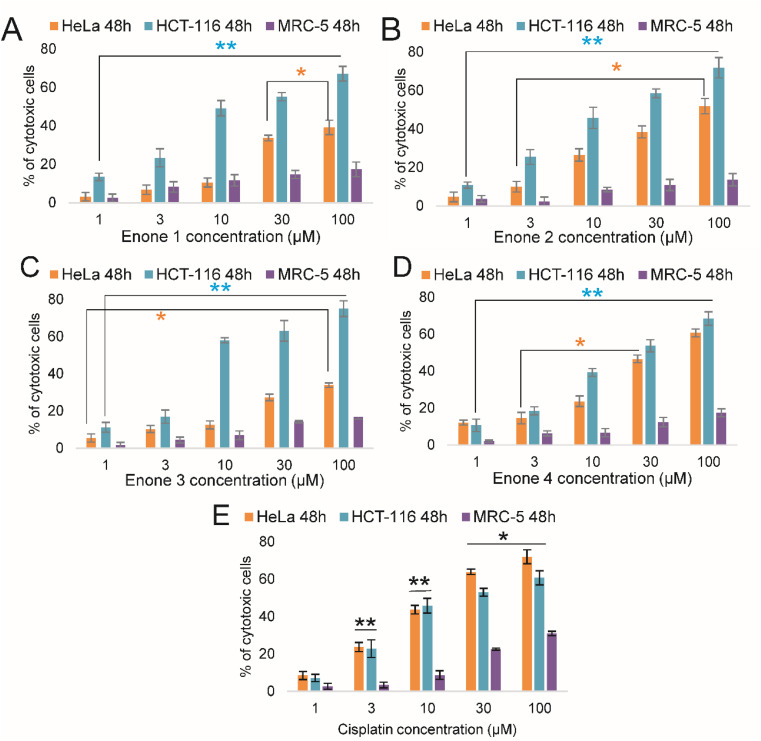
Graphical presentation of cytotoxic effects of enones (E1–E4) applied at different concentrations during the 48 hour towards HeLa, HCT-116, and MRC-5 cells (A–D) and compared to the cytotoxic effect of cisplatin (E). Probability values of (*p* < 0.05* and *p* < 0.01**) were considered statistically significant. Points, mean % of cell cytotoxicity based on quintuplicate assays, bars, ± SE of triplicate experiments.

In order to evaluate and analyze the possible cytotoxic properties of the newly synthesized enones, the MTT assay was used to determine the cytotoxicity in human colon cancer cells (HCT-116), human cervical cancer cell line (HeLa), and human fibroblast lung cell line (MRC-5). Cells were incubated with varying concentrations of enones (1, 3, 10, 30, and 100 μM) for 48 and 72 hours. Our results demonstrated significant cytotoxic effects of all examined enones (*p* < 0.05* and *p* < 0.01) against the cancer cell lines (HCT-116 and HeLa) at both time points (48 and 72 h). However, the cytotoxic effect of enones on the control MRC-5 cell line was considerably less (Fig. 3) with the highest percentage of MRC-5 cells killed only reaching 17. 46% at the highest dose used of Enone 4. Cytotoxicity ranges for 48 h treatment and different concentration ranges (1–100 μM) were for HeLa (E1 = 3.12–39.24; E2 = 4.74–51.94; E3 = 5.47–33.95; E4 = 12.12–60.72 and cisplatin = 8.47–71.99); HCT-116 (E1 = 11.23–72.91; E2 = 10.87–71.88; E3 = 11.23–75.08; E4 = 39.29–68.45 and cisplatin = 7.5–60.72).

Examining the enones' cytotoxic effect, it is evident that, on HCT-116 cells, the effect was the strongest and most statistically significant (*p* < 0.01**), with the order of cytotoxicity being E2 > E1 > E3 > E4 (strongest to weakest cytotoxic effect). Furthermore, compared to control cells, the cytotoxic effect of enones on HCT-116 cells was statistically significantly greater (*p* < 0.01**). Enones had notable cytotoxic effects on HeLa cells, albeit they were less pronounced (*p* < 0.05*) when compared to the control. These findings suggest that colon cancer cells (HCT-116) were more sensitive to the examined enones' cytotoxic effects than cervical cancer cells (HeLa).

Specifically, comparing the percentage of cytotoxic HCT-116 cells to control cells (MRC-5), treatment with enone 1 (at different doses) significantly increased (4.1–4.4-fold). Enone 1's cytotoxic effect on HCT-116 cells was, at all applied doses, 1.8–3.6 times greater than that of HeLa cells. Enone 2 raised the percentage of cytotoxic HCT-116 cells by 1.3–2.3-fold when compared to HeLa cells and by 2.9–5.2-fold compared to MRC-5 cells. The proportion of cytotoxic HCT-116 cells was elevated by Enone 3 relative to HeLa cells, 2.05–2.1 times, and to control MRC-5 cells, 4.4–6.9 times (Fig. 3A–D). Cisplatin exhibited statistically significant cytotoxic effects (*p* < 0.01**) on HeLa and HCT-116 cells at doses of 3 and 10 μM, as well as *p* (<0.05*) at enones dosages of 30 and 100 μM compared to control MRC-5 cells. Cisplatin produced a lower cytotoxic effect on HCT-116 cells than any other enone tested, but a larger cytotoxic effect than enone-treated HeLa cells. However, when the cytotoxic effects of cisplatin and enones were compared, cisplatin had a greater lethal effect on MRC-5 cells, indicating that enones were more selective for cancer cells than cisplatin (Fig. 3E).

**Fig. 4:**
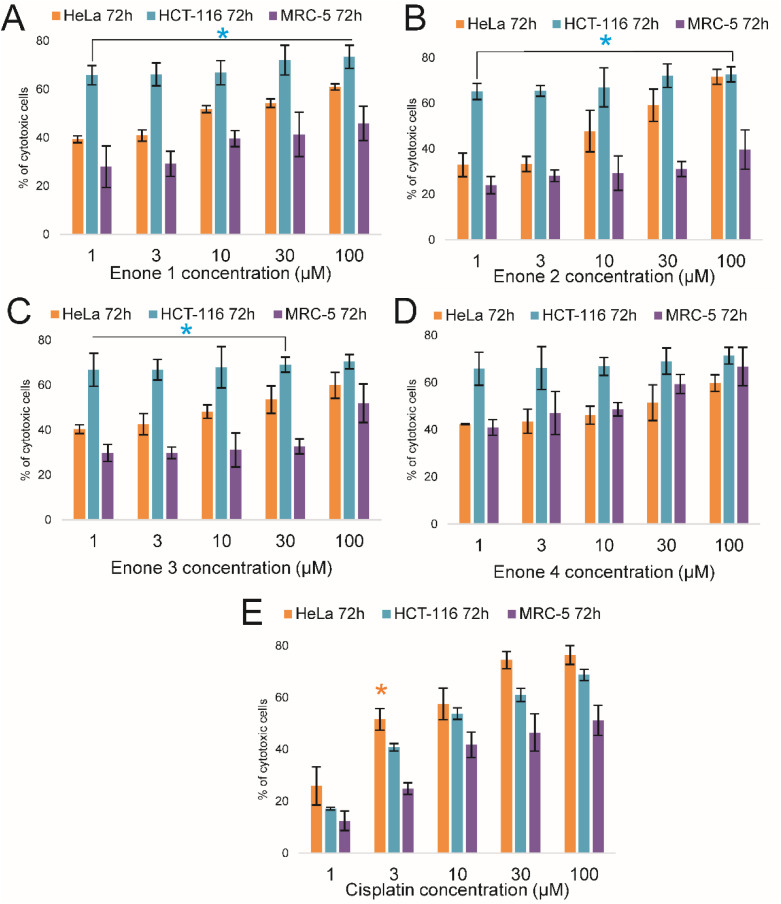
Graphical presentation of cytotoxic effects of enones (E1–E4) applied at different concentrations during the 72 hour towards HeLa, HCT-116, and MRC-5 cells (A–D) and compared to the cytotoxic effect of cisplatin (E). Probability values of (*p* < 0.05* and *p* < 0.01**) were considered statistically significant. Points, mean % of cell cytotoxicity based on quintuplicate assays, bars, ± SE of triplicate experiments.

Compared to 48 hours of treatment, the enones' cytotoxic effect on HeLa cells greatly increased after 72 hours. Cytotoxicity range for 72 h period for HeLa (E1 = 39.35–61; E2 = 32.98–71.66; E3 = 40.39–59.92; E4 = 42.39–59.81 and cisplatin = 25.91–76.35); HCT-116 (E1 = 65.85–73.44; E2 = 65.23–72.73; E3 = 66.85–70.44; E4 = 65.85–71.44 and cisplatin = 17.15–68.72).Concurrently, the enones persisted in a cytotoxic tendency on HCT-116 cells (Fig. 4A–D). Compared to 48 hours of treatment, the enones' cytotoxic effect on HeLa cells greatly increased after 72 hours. Concurrently, the enones persisted in a cytotoxic tendency on HCT-116 cells (Fig. 4A–D). The MTT assay results revealed that the most effective sequence of enones for causing cytotoxicity in cancer cells was E2 > E1 > E3 > E4 after 72 hours. On HCT-116 cells, E1, E2, and E3 demonstrated statistically significant (*p* < 0.05*) cytotoxic effects. Remarkably, these enones had similar cytotoxic effects on HeLa and HCT-116 cells at the maximal concentration of 100 μM.

### 3.2. IC_50_ values and selectivity index

Table 1 displays the calculated IC_50_ values of enones (E1–E4) on all cell lines tested at 48 and 72 hours. The computed IC_50_ values for 48 hours of treatment showed that all enones produced lower IC_50_ values against HCT-116 (5.1–9.4 μM) than against HeLa cells (13.3–20.1 μM) and cisplatin-treated cells (13.6 and 16.5 μM). IC_50_ values for enones in MRC-5 cells exceeded 1_50_ μM (E1), 200 μM (E2), and 100 μM (E3, E4) after 48 hours. After 72 hours of treatment, all investigated enones had reduced IC_50_ values in all tested cells.

**Table 1:** Calculated IC_50_ values of E1, E2, E3, and E4 in examined cells following 48 and 72 h of treatment. The IC_50_ values are represented as the mean ± SD of triplicate experiments

	IC_50_ (μM)					
	48 h			72 h		
	HeLa	HCT-116	MRC-5	HeLa	HCT-116	MRC-5
E1	17.31 ± 1.19	6.06 ± 0.96	>150	3.59 ± 0.03	1.12 ± 0.01	>65
E2	13.37 ± 1.43	5.10 ± 0.92	>200	3.43 ± 0.09	0.9 ± 0.02	>90
E3	18.19 ± 1.33	6.8 ± 1.01	>100	3.96 ± 0.1	1.64 ± 0.01	>70
E4	20.13 ± 1.19	9.42 ± 1.78	>100	4.12 ± 0.08	2.0 ± 0.01	>80
CP	16,5 ± 1.11	13,6 ± 0.34	>30	3.62 ± 0.09	7.08 ± 0.13	>85

Next, the Selectivity Index (SI) was calculated and presented in Table 2 to assess the therapeutic potential of the investigated enones. The results showed that E2 had the highest SI in HCT-116 and HeLa cells after 48 and 72 hours. The selectivity index of all analyzed enones was high at all tested time intervals and in cancer cells due to the high IC_50_ values for MRC-5 cells.

**Table 2:** Selectivity index of E1–E4 for HCT-116 and HeLa cells. The SI values are represented as the mean ± SD of triplicate experiments

	Selectivity index (SI)			
	48 h		72 h	
	HeLa	HCT-116	HeLa	HCT-116
E1	8.67	24.75	19.18, 18.10	58.03
E2	14.96	39.22	26.23	100.63
E3	5.50	14.71	17.67	42.68
E4	4.97	10.62	19.41	40.00
CP	1.88	2.28	23.48	12.00

### 3.3. Newly synthesized enones, E1–E4, induced morphological changes in HCT-116 and HeLa cells

**Fig. 5:**
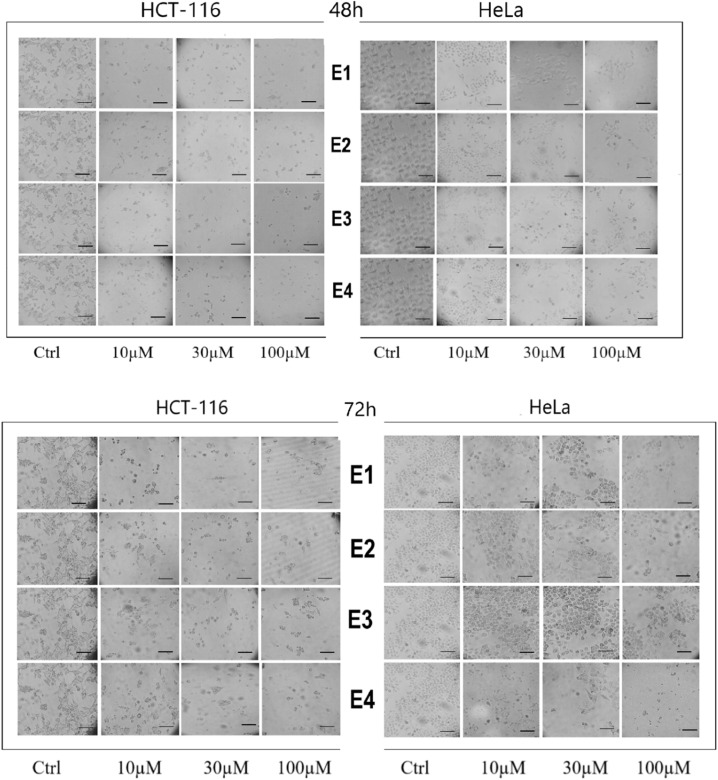
Morphology of HeLa and HCT-116 cells after 48 and 72 hours of incubation with various doses of enones (E1–E4). Morphology of tumor cells was investigated using phase-contrast microscopy. Enone treatment altered the morphology of cancer cells compared to the control (scale bar 100 μm).

Cancer cells treated with enones (10, 30, and 100 μM) for 48 hours showed morphological alterations due to their cytotoxic effects. Morphological investigation of enones-treated cells indicated changes in cell morphology, including membrane blebbing, loss of cell attachment, and rounding. Following the apparent loss of normal cell shape, attachment and contact with adjacent cells were disrupted. The number of viable cells was clearly decreased by enone therapy among all other morphological changes. The morphological changes were time and dose-dependent (Fig. 5). Morphological alterations in treated cells after 72 hours revealed clear disruption of cell structure.

### 3.4. Newly synthesized enones induced apoptosis in HCT-116 and HeLa cells

**Fig. 6:**
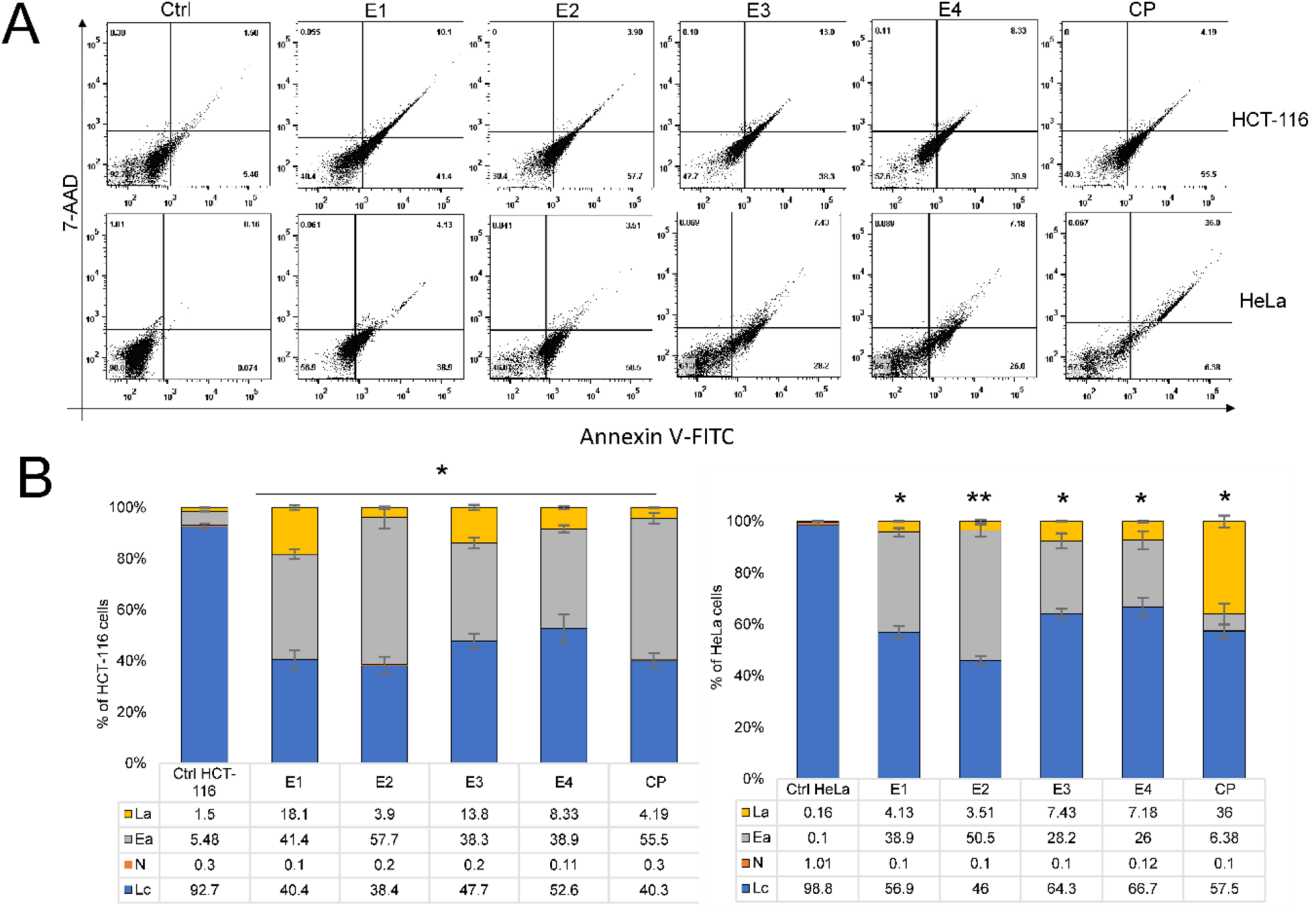
The apoptotic effect of enones on HCT-116 and HeLa cells after 48 hours. (A) The percentage of apoptotic HCT-116 and HeLa cells following enones treatment is presented by dot plots. (B) Bar graphs show the percentage of HCT-116 and HeLa cells treated with enones relative to untreated, control cells (La-late apoptotic, Ea-early apoptotic, N-necrotic and Lc-live cells). Bar graphs present percentage of cells (live or dead) *vs.* IC_50_ (μM) values of E1–E4 and cisplatin used of readings from triplicate experiments; bars, ± SEM; *p* < 0.05*, and *p* < 0.01** *vs.* the control group (Ctrl).

Next, we analyzed the type of cell death triggered by enones in cancer cells based on their cytotoxic and selective effect on HCT-116 and HeLa. Enones induced apoptosis in cancer cells, which promoted cell death, as seen by the Annexin/7AAD staining results (Fig. 5). The highest percentages of apoptotic tumor cells treated with various enones (IC_50_ values-Table 1) were detected in the early phases of apoptosis, showing that the initial phase of cell death had been induced. The percentage of total apoptotic HCT-116 cells (the sum of early and late apoptotic cell percentages) was statistically significant (*p* < 0.05*) after 48 hours of enone treatment in comparison to control cells (E1-59.5%, E2-61.6%, E3-51.68%, E4-47.23%, and CP-59.69%). The percentage of apoptotic HCT-116 cells treated with enones increased statistically significantly (E1 – 8.5-fold), (E2 – 8.8-fold), (E3 – 7.4-fold), and (E4 – 6.7-fold) as compared to the control HCT-116 cells. The findings also demonstrated that the percentage of total apoptotic enone-treated HeLa cells was lower when compared to the percentage of enone-treated apoptotic HCT-116 cells. The total percentage of apoptotic HeLa cells was 43.03% for E1, 54.02% for E2, 35.63% for E3, 33.18% for E4, and 42.38% for CP. The percentage of apoptotic cells in enones-treated HeLa cells was significantly (*p* < 0.05*) higher than that of control HeLa cells. The results for HeLa and HCT-116 cells showed that E2 > E1 > E3 > E4 was the most effective apoptotic sequence. These data suggest that enones exerted cytotoxicity in HC116 and HeLa cancer cells by triggering apoptosis (Fig. 6).

### 3.5. Newly synthesized enones regulate expression of Bax and Bcl-2

**Fig. 7:**
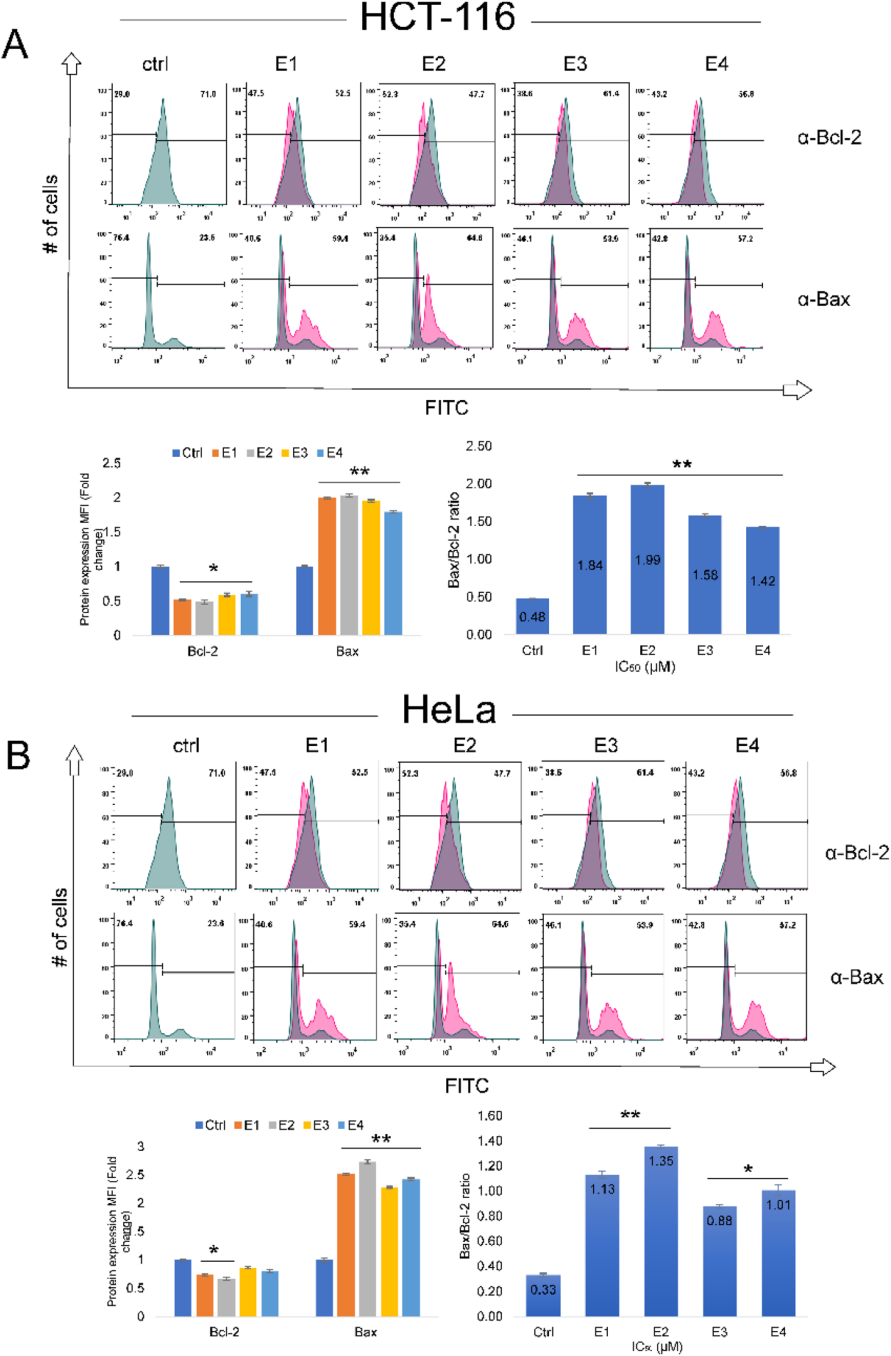
E1–E4 induce upregulation of Bax and downregulation of Bcl-2 in HCT-116 and HeLa cells. Bax/Bcl-2 ratio was measured in HCT-116 and HeLa cells after 48 hours of treatment with different enones (IC_50_ values) compared to control cells. (A) Flow cytometry histogram analysis of the expression of key apoptotic regulatory proteins (Bcl-2 and active Bax) followed with bar graph for protein expression MFI (fold change) and Bax/Bcl-2 ratio in HCT-116 cells. (B) Flow cytometry histogram analysis of the expression of key apoptotic regulatory proteins (Bcl-2 and active Bax) followed with bar graph for protein expression MFI (fold change) and Bax/Bcl-2 ratio in HeLa cells. Bars represent the mean percentage of cell distributions from three independent experiments: bars, ± standard error (*p* < 0.05* and *p* < 0.01** *vs.* the control group (Ctrl)).

**Fig. 8:**
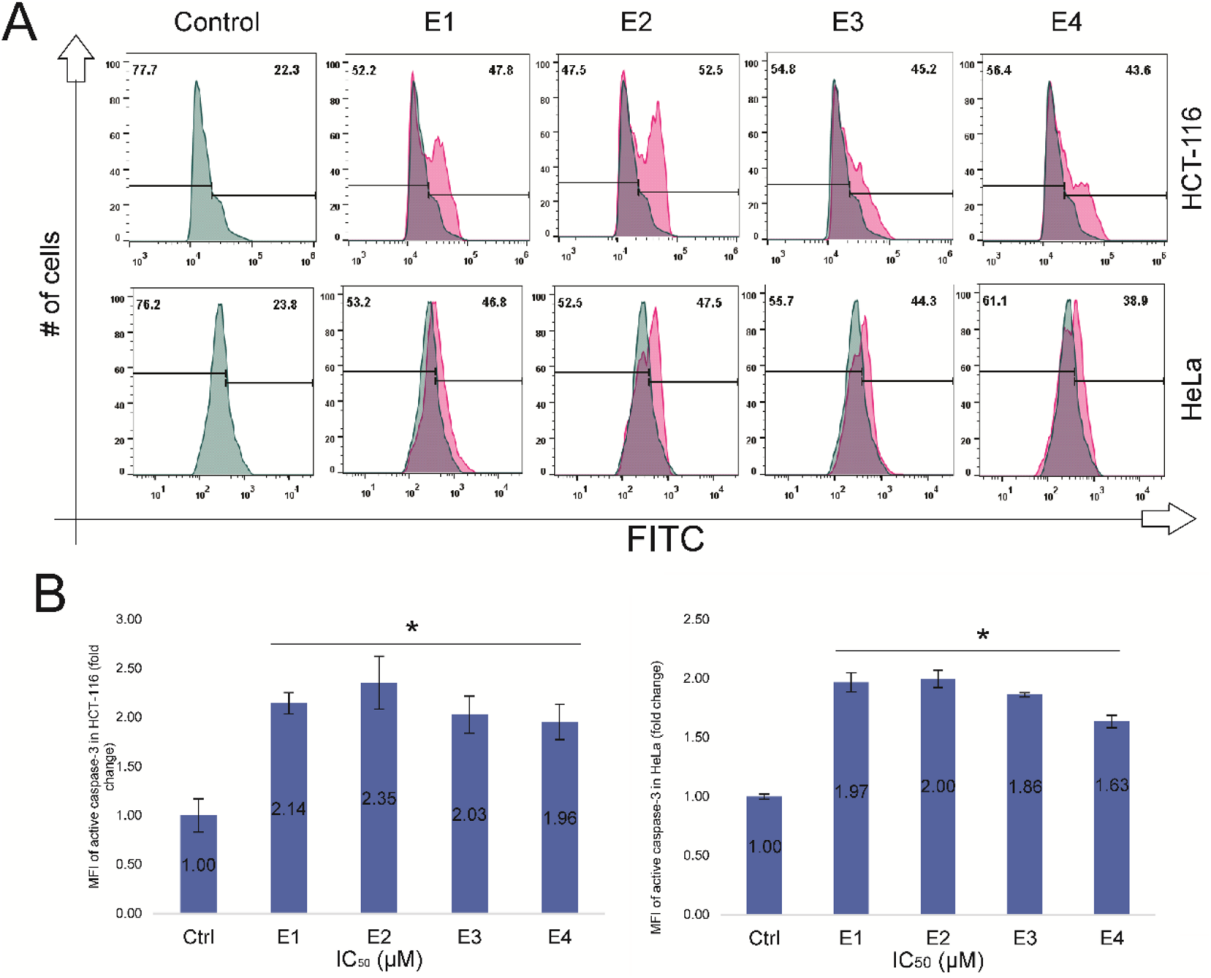
E1–E4 induce the activation of caspase 3 in HCT-116 and HeLa cells. (A) Flow cytometry histogram analysis and bar graph of the expression active caspase-3 in HeLa and HCT-116 after enones treatment (E1–E4). (B) Bar graph presentation of MFI of active caspase-3 (fold change) in HCT-116 and HeLa cells. Bars represent the mean percentage of cell distributions from three independent experiments: bars, ± standard error (*p* < 0.05* and *p* < 0.01** *vs.* the control group).

Since enones increased the proportion of cancer cells undergoing early apoptosis, we investigated how they affected the expression levels of important regulatory apoptotic proteins: Bax, Bcl-2, and executioner caspase-3 (Fig. 7 and 8). We applied FACS analysis to examine the influence of enones (IC_50_ values- Table 1) on the expression of Bax and Bcl-2 proteins, as well as their fold change in treated cancer cells. The expression of the pro-apoptotic Bax protein was significantly upregulated (*p* < 0.01**) in HCT-116 cells subjected to IC_50_ values of enones (E1–E4) for 48 hours, whereas the antiapoptotic Bcl-2 protein was downregulated (*p* < 0.05*) in comparison to control cells (Fig. 7A). Enones E1–E4 significantly enhanced the Bax/Bcl-2 ratio in HCT-116 cells (1.8, 1.9, 1.6, and 1.4, respectively; *p* < 0.01**). In HeLa cells, E1 and E2 significantly (*p* < 0.05*) decreased Bcl-2 protein expression, while all tested enones significantly increased Bax protein (*p* < 0.01**) compared to control cells (Fig. 7B). Analysis of the fold increase in the Bax/Bcl-2 ratio between enones-treated and control HeLa cells revealed significant differences (*p* < 0.01**) for E1 and E2 (1.1 and 1.4, respectively), and significant changes (*p* < 0.05*) for E3 and E4 (0.9 and 1-fold). Pro-apoptotic Bax overexpression and antiapoptotic Bcl-2 downregulation are two main hallmarks of the intrinsic apoptotic pathway's activation.

### 3.6. Newly synthesized enones caused activation of caspase-3 in HCT116 and HeLa cells

In the next experiment, we evaluated the level of active caspase-3 expression and compared fold changes in caspase-3 expression between enones-treated and untreated tumor cells. Enones treatment (IC_50_-48 hour – Table 1) significantly elevated active caspase 3 expression in HCT-116 and HeLa cancer cells (*p* < 0.05*). In HCT-116 cells, treatment with various enones (E1–E4) resulted in a 2-fold increase in active caspase-3 expression compared to control cells (E1-2.1; E2-2.4; E3-1.8, and E4-1.9); in HeLa cells, this fold increase was equally significant (E1-1.9; E2-2; E3-1.8, and E4-1.6) compared to control. These findings suggest that the newly synthesized enones triggered apoptosis in HCT-116 and HeLa cells by downregulating Bcl-2, upregulating Bax. and activating caspase-3. It is noteworthy that HCT-116 cells treated with enones exhibited a statistically significant larger increase in active caspase-3 expression when compared to HeLa cells (Fig. 8A and B).

### 3.7. Newly synthesized enones cause dysregulation of Δ*Ψm* in tumor cells

**Fig. 9:**
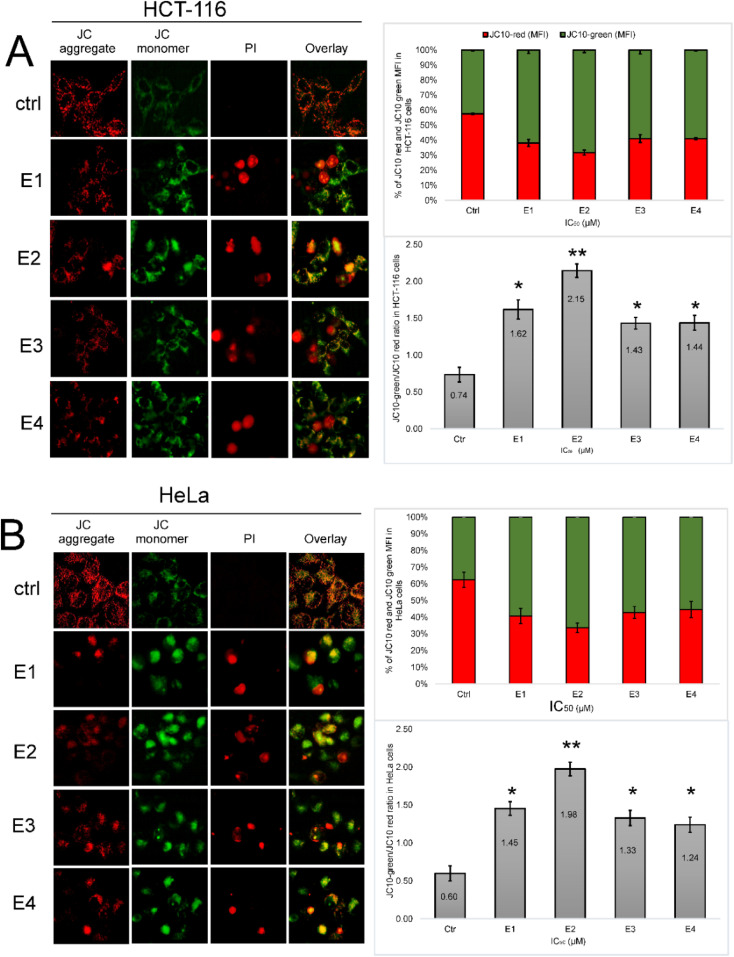
Effects of different enones on mitochondrial membrane potential (Δ*Ψm*) in tumor cells shown by mitochondrial fluorescence probe JC-10. Immunofluorescence observations of JC-10 in control compared to enones-treated tumor cells ((A) HCT-116 and (B) HeLa) and their quantitative results (presented as bar graphs) of monomer/aggregate ratio were assumed to be proportional to Δ*Ψm* intensity. Bars represent the mean percentage of cell distributions from three independent experiments: bars, ± standard error (*p* < 0.05* and *p* < 0.01** *vs.* the control group) (scale bar 20 μm).

To establish the role of mitochondria in enones-induced cell death, we next investigated the impact of enones on tumor cells' mitochondrial membrane potential. Cancer cells were treated with enone IC_50_ values (Table 1) for 48 hours. Using the JC-10 dye and immunofluorescence methods, we examined the changes in the mitochondrial membrane potential of the enones-treated cells (Fig. 8). The results demonstrated that enone treatment decreased Δ*Ψm* in HCT-116 and HeLa cells. The percentage of red JC-10 aggregate-containing cells (control cells) in HCT-116 (Fig. 9A) and HeLa (Fig. 9B) was almost 60%, but in enone-treated HCT-116 and HeLa cells, that percentage was approximately 30% and 40%, respectively. These findings suggested that enone administration induced JC-10 to diffuse from mitochondria to the cytosol. The intensity of JC-10 red fluorescence was highest in control HCT-116 and HeLa cells, while green fluorescence intensity (MFI) was low. Enone treatment of cancer cells disrupted Δ*Ψm*, leading to enhanced cytosolic JC-10 green fluorescence. Treatment of tumor cells with enones IC_50_ values for 48 hours significantly increased (*p* < 0.05*) fold ratio of JC-10 green/JC-10 red ratio for E1, E3, and E4 (1.6; 1.4 and 1.4 respectively) and (*p* < 0.01**) for E2 (2.15) in HCT-116 cells (Fig. 9A). In HeLa cells, the fold change of JC-10 green/JC-10 red ratio for E1, E3, and E4 (1.4; 1.3 and 1.2 respectively) was statistically significant (*p* < 0.05*) with E2 showing a fold change in JC10 green/red ratio of 1.9 (*p* < 0.01**) (Fig. 9B). The enone-induced Δ*Ψm* alterations for both tumor cell lines were graded (strongest to weakest) as follows: E2 > E1 > E3 > E4.

### 3.8. E1–E4 trigger translocation of cytochrome c from mitochondria to the cytosol in tumor cells

**Fig. 10:**
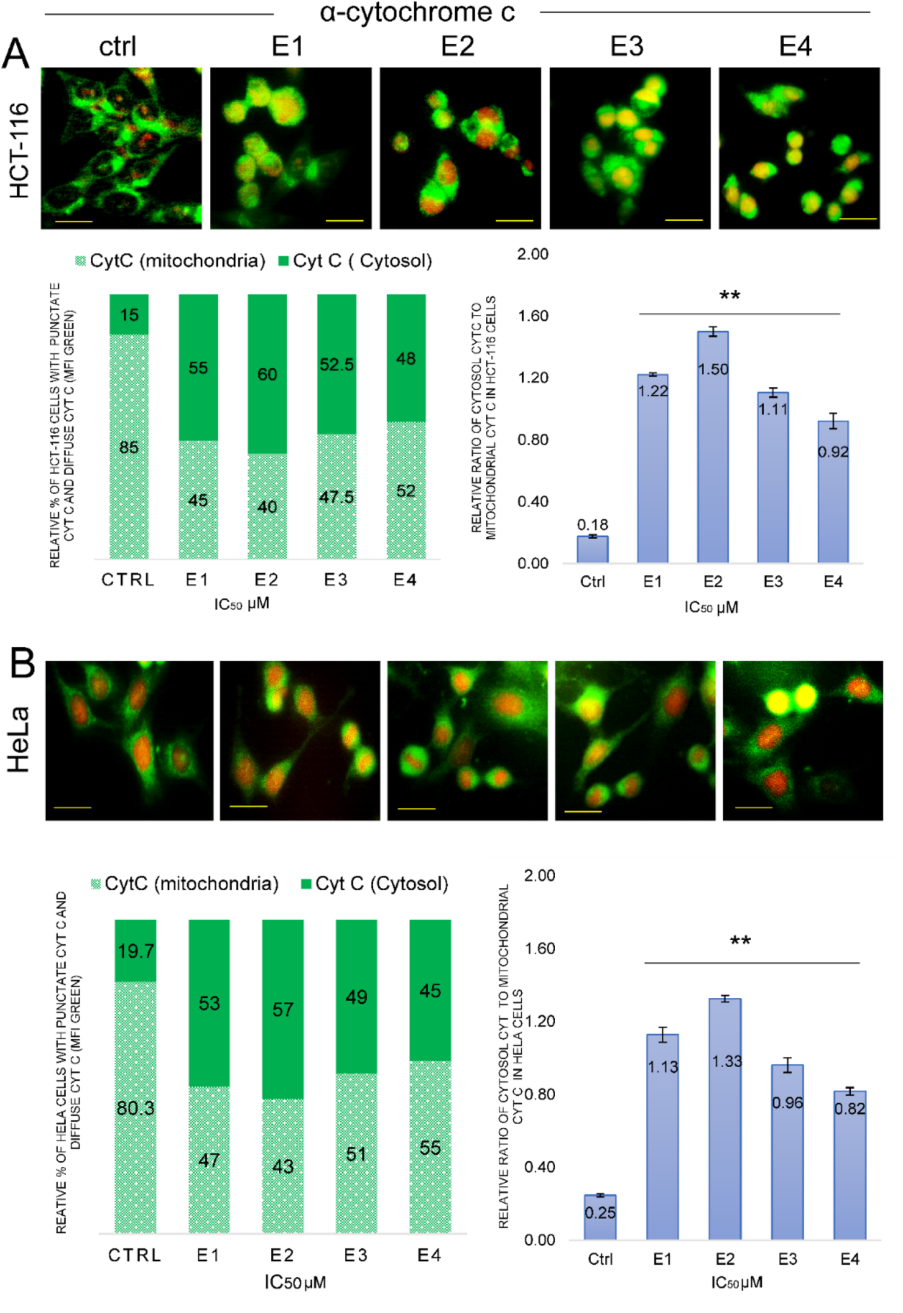
Induction of cytochrome c release from the mitochondria in HCT-116 and HeLa cells following 48 h treatment with examined enones (E1–E4). Immunofluorescence analysis of cytochrome c in the mitochondria is shown in control and enones (E1–E4) treated HCT-116 (A) and HeLa (B) cells. Images were analyzed using ImageJ software and results were presented by bar graphs (scale bar 50 μm). Bar graphs show the mean % of cells with mitochondrial (green diffuse) and cytosol (green punctate) localization of cytochrome c (*p* < 0.01** compared to the control).

Next, we assessed the expression levels and localization of cytochrome c in cancer cells exposed to enones using immunofluorescence assay (Fig. 9). Cytochrome c was significantly (*p* < 0.01**) translocated from the mitochondria to the cytoplasm in both HeLa and HCT-116 cells following enones treatment. In HCT-116 cancer cells, enones administration significantly raised the ratio of cytochrome c in the cytosol to mitochondria (*p* < 0.01**) compared to the control (E1-1.2, E2-1.5, E3-1.1, and E4-0.9). The relative fold change in cyt c cytosol/cyt c mitochondria ratio also increased by enone treatment of HeLa cells, however, the rise was greater in HCT-116 cells (Fig. 10).

### 3.9. Newly synthesized enones induced G2/M cell cycle arrest in tumor cells

**Fig. 11:**
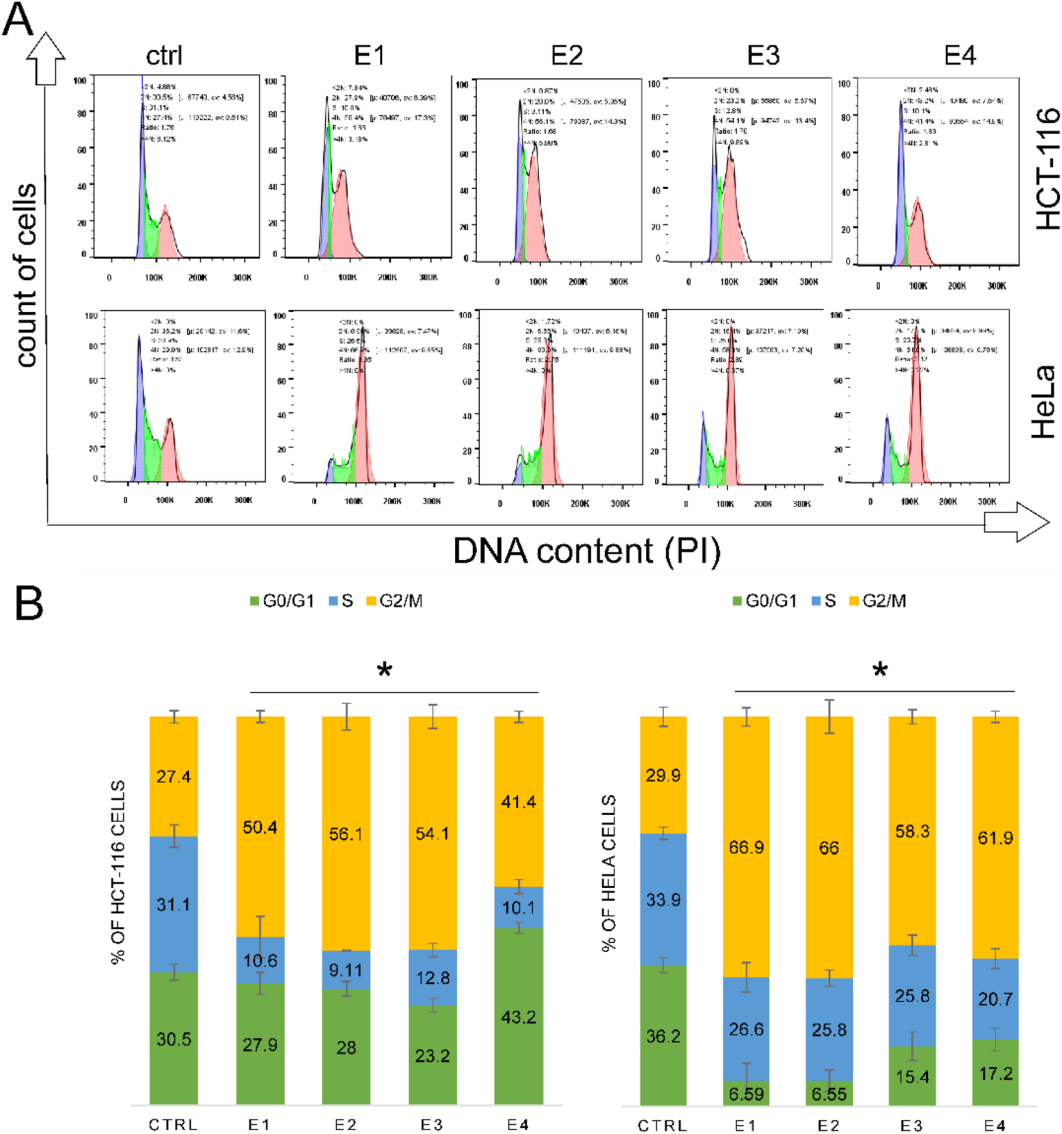
Enones induce G2/M cell cycle arrest in HCT-116 and HeLa cells. Enones (E1–E4) caused cell cycle arrest in the G2/M phase in the tumor cells. Tumor cells were treated with IC_50_ values of each enone for 48 hours, after which the cells were collected, fixed, and stained with PI for FACS analysis. (A) The cell cycle profiles of the enones-treated tumor cells were analyzed by flow cytometry and the percentages of cells distributed across different phases of the cell cycle were calculated using the FlowJo software. (B) These results are presented as histogram bars representing the mean percentage of cell distributions from three independent experiments (bars, ± standard error, *p* < 0.05* compared to the control group).

Using flow cytometry analysis, we further examined the impact of enones on cell cycle arrest in cancer cells. In both HCT-116 and HeLa cells, enones administration resulted in a significant (*p* < 0.05*) G2/M phase cell cycle arrest (Fig. 11). Enone 2 was the most effective at accumulating HCT-116 cells in the G2/M phase (56.1%), compared to other enones (E1-50.4%, E3-54.1%, and E4-41.4%) and control cells (27.4%). The most effective sequence for G2/M phase cell cycle arrest in HCT-116 cells was E2 > E3 > E1 > E4. The findings indicate that enones may cause growth inhibition and cell death in colon cancer cells through a variety of mechanisms, including G2/M arrest. Enones administration caused G2/M phase accumulation in HeLa cells, although with a distinct efficiency sequence (E1 > E2 > E4 > E3). In HeLa cells, the most effective enones to trigger the G2/M cell cycle arrest were E1 (66.9%), E2 (66%), E4 (61.9%), and E3 (58.3%) compared to the control of 29.9%.

## 4 Discussion

Cancer research and treatment are among the most extensively studied areas of biomedical and molecular research. Each year, the number of patients diagnosed with carcinomas continues to rise. Colon and cervical cancer are among the top five cancers with rising prevalence rate worldwide. Therapeutic options for treating these tumors are expanding, with a greater emphasis on minimizing adverse effects for patients. The incidence of all cancer types, across both sexes, has grown over the past two to three decades.^11^

In this study, we synthesized four structurally novel enones (E1–E4) that differ only in one substituent and then investigated their antitumor potential against HCT-116 and HeLa cells.

Specifically, enone 1 possesses a methyl group, E2 has an isopropyl group, E3 contains an isobutyl group, and E4 comprises a cyclopropyl group (derived from vanillin). Vanillin, a naturally occurring chemical, served as the foundation for the synthesis of these enones.

Tseng *et al.*,^12^ discovered that by substituting the quinoline ring with a C-6 methoxy group in compound (*E*)-6-methoxy-3-(4-methoxyphenyl)-2-[2-(5-nitrofuran-2-yl)vinyl]quinoline 22 inhibited metastasis and had a strong cytotoxic effect in A549, H1299, MCF-7, and MDA-MB-231 cell lines. It has been proven that enhancing a drug's primary anticancer efficacy by adding a different chemical substituent can be beneficial in the treatment of cancer 1. For instance, adding methyl substituents to oxaliplatin produced enantiomerically pure oxaliplatin analogs, which enhanced the therapeutic properties of the parent medication while drastically lowering side effects.^13,14^ It has been discovered that isopropyl groups improve anticancer drug selectivity^15^ and exhibit various biological activities over the original compounds.^16^ Moreover, the addition of an isobutyl group to the imidazolidin-2-one (IMZ) moiety produced anti-proliferative activity and selectivity for breast cancer cell lines (MCF7 and MDA-MB-468) cell lines.^17^ Other studies also demonstrated guanidinium's anticancer properties against HepG2, MCF-7, and SW480.^18^

Barbosa *et al.*,^19^ found that dimethoxy substituents had a stronger inhibitory effect on EGFR and VEGFR-2, which play important roles in apoptosis, cell proliferation, and migration. This result, along with the findings of Wu *et al.*,^20^ emphasizes the importance of methoxy groups in the antimetastatic efficacy of anticancer drugs. The methoxy substituent present in all newly synthesized enones used in this research has been shown to exhibit anticancer activity, which is one of the reasons it was included in this study. The synthesis of the enones examined in this study relied primarily on the presence and significance of different alkyl groups (methyl, isopropyl, isobutyl, and cyclopropyl) connected to the carbonyl group. Another natural compound, dehydrozingerone, can be synthesized by starting with the basic molecule vanillin. Dehydrozingerone exhibits a variety of pharmacological actions, including anti-inflammatory, anti-depressive, antibacterial, antiviral, and anticancer activities.^21^

Researchers identified the presence of a methyl group connected to a carbonyl group as a critical feature in its biological activity, resulting in the synthesis of the compound E1 from vanillin, which was used in this study. Long *et al.*,^22^ reported that compounds containing isopropyl and isobutyl groups inhibited HCT-116 and NCI-H460 cells, with IC_50_ values ranging from 30 to 80 μM. Similarly, De *et al.*,^23^ demonstrated that their novel 2 (4-alkoxyphenyl) cyclopropyl hydrazide derivatives exhibited antiproliferative effects on U373, A549, SKMEL-28, OE2, PC-3, and MCF-7 cells.^24^ discovered that analogues with the following potency hierarchy: 7-cyclopropyl- > 7-propyl- > 7-butylacetylene, demonstrated high cytotoxic activity with IC_50_ values of 60 μM against a panel of human hematological and solid cancer cell lines.

Our findings were consistent with those reported in previous investigations.^9^ Our four newly synthesized enones (E1–E4) showed significantly reduced IC_50_ values (5.1–9.4 μM) and increased IC_50_ values for healthy cells (>100 and 200 μM) after 48 hours of treatment, compared to earlier studies with various enones. Enones' cytotoxic effect increased after 72 hours, although it was also accompanied by a larger cytotoxic effect on healthy cells. Following a 48 hour therapy, each of the four enones showed significant anticancer potency against both HeLa and HCT-116 cells (*p* < 0.05* and *p* < 0.01**—respectively). Similar outcomes were previously reported by Luković *et al.*,^9^ using the other newly synthesized vanillin analogs.

The selectivity index of our newly synthesized enones (E1–E4) showed strong selectivity toward tumor cells, with overall greater cytotoxic and selective potencies directed toward colon cancer cells compared to cervical cancer cells. Several investigations have found that different enones exhibited a high SI on various types of cancer cells. Roayapalley *et al.*,^25^ discovered that their 5-diaryl-3-oxo-1,4-pentadienes, mounted on a piperidine ring based on a substituted group, demonstrated a selective anticancer effect against human HT29 colorectal adenocarcinoma and human squamous carcinoma-4 (HSC-4). Pati *et al.*,^26^ similarly reported that 1,3-diaryl-2-propenones and 2-benzylidene-1,3-indandiones were more cytotoxic to neoplastic cells than control cells. The cytotoxic effect of many potential antitumor agents inevitably affects cell morphology. Malignant and healthy cells have different morphologies, and during the normal cell cycle and in response to internal and external stressors, alterations in cell morphology are inevitable. According to Scipioni *et al.*,^27^ novel vanillin compounds induced apoptosis and exhibited morphological alterations characteristic of apoptotic cells. Numerous other investigations also demonstrated that enones or vanillin analogs can alter the morphology of different types of tumor cells. For example, Ca9-22 cells exhibited shrinkage, rounding, and the formation of apoptotic bodies 24 hours after being treated with 1,5-diaryl-3-oxo-1,4-pentadienes linked to a piperidine ring.^25^ In our study, the enones (E1–E4) generated dose-dependent morphological alterations in HeLa and HCT-116 cells, which were consistent with changes observed in dying cells, as compared to untreated control cells. Notable morphological alterations in cells including loss of cell attachment, and apoptotic bodies were also observed. HeLa cells showed less significant morphological alterations compared to HCT-116 cells. Enones treatment had the least impact on the morphology of the healthy control cell line (MRC-5).

The cytotoxic effects of various substances may trigger cell death by a variety of pathways, including apoptosis, necrosis, endoplasmic stress, necroptosis, and others. Our results demonstrated that after 48 hours of enones treatment, the percentage of total apoptotic cells in HCT116 and HeLa tumor cells increased significantly (*p* < 0.05*). Da'I *et al.*^28^ discovered that pentagamavunon and curcumin induced apoptosis in HeLa, T47D, and MCF-7 tumor cells.

Consistent with results from prior investigations, our data demonstrated a significant apoptotic effect of enones. For instance, Gurung *et al.*'s^29^ demonstrated that thymoquinone (TQ) promoted apoptosis in M059K glioblastoma cells by activating the p53-independent pathway and increasing Bax and cytochrome c protein levels. Zhang *et al.*^30^ showed that TQ exposure caused growth inhibition and apoptosis in bladder cancer cell lines (T24 and 253). The potential anticancer mechanism of TQ involved changes in the expression of Bcl-2, Bax, cytochrome c, and endoplasmic reticulum stress-related proteins, indicating the link between mitochondrial dysfunction and the endoplasmic reticulum stress pathway. Oridonin, a natural enone, has been identified as an effective anticancer agent, inducing apoptosis *via* the intrinsic mitochondrial apoptotic pathway in various cell lines, including prostate (LNCaP, DU145, PC3), breast (MCF-7, MDA-MB231), non-small cell lung (NSCL) (NCI-H520, NCI-H460, NCI-H1299), acute promyelocytic leukemia (NB4), and glioblastoma multiforme (U118, U138).^31^

Another study demonstrated that Soloxolone methyl inhibited proliferation and had an anticancer effect on pulmonary adenocarcinoma cells and a cervix cell line, which was mediated *via* apoptosis induction.^32^ Furthermore, synthetic cyan enone derivatives have been shown to induce cell death in a variety of cell lines, including prostate cancer (PPC-1, Alva-31, DU145, LNCAP, and PC3) and glioma cells (U87, U343, U251, LN229, and C6).^33^ The findings obtained in the present study suggested that enones played a significant role in upregulating Bax and downregulating Bcl-2 in colon and cervical cancer cells. In colon cancer HCT116 cells, E1–E4 exhibited a greater effect on the dysregulation of apoptotic proteins Bax and Bcl-2 than in HeLa cells. The dysregulation of the balance of the antiapoptotic and pro-apoptotic proteins is a reversible step in cell death induction. However, all subsequent events in the apoptotic signaling cascade cause irreversible alterations in the cell's energy balance, including changes in Δ*Ψm*, cytochrome c release, and the activation of executioner caspase-3. When apoptosis is induced, Bax oligomerization produces pores in the mitochondrial membrane, making it porous and permeable to different molecules. In this study, the administration of E1–E4 to HeLa and HCT-116 cells produced the significant changes in mitochondrial membrane potential (Δ*Ψm*), indicating that enones induced disruption of Δ*Ψm* in cancer cells. Several investigators have also reported effect of vanillin, curcumin, and chalcone derivatives on mitochondrial dysfunction and subsequent cytochrome C release from mitochondria in various cancer cells.^34–38^ Cytochrome c, a well-studied protein, is a reliable marker for mitochondrial apoptosis, and is localized in the intermembrane space of mitochondria.^39^ Following the apoptotic stimuli, changes in the mitochondrial membrane cause cytochrome C to translocate into the cytoplasm, leading to in the formation of apoptosome and the subsequent activation of caspase 3, which results in the execution of apoptosis.^40^ Research has been done extensively on a variety of cell types regarding the induction of apoptotic cell death by enones or derivatives containing enones.^41,42^ Our findings are consistent with those of other authors, demonstrating that the newly synthesized enones (E1–E4) induced the release of cytochrome c into the cytoplasm of treated tumor cells, driving the apoptotic process to its final execution phase. The cell cycle is a strictly regulated process that occurs in all cell's types, and various substances can cause the arrest of the cells at certain phases of the cycle. This arrest disrupts the typical duration and phases of the cell cycle, leading to the cell growth inhibition and death. In this study, E1–E4 induced cell cycle arrest in the G2/M phase in HCT-116 and HeLa cancer cells. Previous studies demonstrated that chalcones caused cell cycle arrest in the G1/S phase,^43^ vanillin derivates in the G0/G1 phase,^44,45^ and dehydrozingerone and curcumin in the G2/M and G1 phases.^46,47^ In conclusion, our research revealed that all newly synthesized enones investigated in this study, were significantly and selectively cytotoxic to HCT-116 and HeLa cells. The observed morphological alterations in enones-treated cells were dose-dependent and associated with apoptotic changes. Enones-induced apoptosis in cancer cells was characterized by downregulation of antiapoptotic Bcl-2 and overexpression of pro-apoptotic Bax. In addition, the newly synthesized enones (E1–E4) caused the G2/M cell cycle arrest, which inhibited the proliferation of tumor cells. On the other hand, the loss of mitochondrial membrane potential led to the release of cytochrome c and the activation of caspase 3 leading to the execution of apoptosis in HCT116 and HeLa cells.

## 5 Conclusion

Our findings indicated that enones (E1–E4) may be promising antitumor candidates for future *in vivo* investigations on colon cancer due to their cytotoxicity, efficiency, and selective antitumor impact, particularly when compared to cervical cancer cells. The newly synthesized enones demonstrated a significant anticancer effect on HCT-116 colon cancer cells, without exhibiting cytotoxic effects on healthy control cells. These results suggested that enones exerted a selective anticancer effect against colon cancer cells. This compelling outcome warrants further investigation in our future *in vivo* studies. The study's findings also demonstrated that, in comparison to healthy control cells, enones significantly reduced the growth of cervical cancer cell lines. Future research on the impact on cervical cancer cells should not be disregarded, even though the main focus may be on colon cancer.

## Data availability

All data included and leading to conclusion presented in this manuscript are included in the ESI.†

## Author contributions

M. B.: writing – original draft, investigation; I. N. investigation, visualization, original draft; review & editing, M. M. software, visualization, resources; investigation; J. M. and T. T. resources and synthesis M. A. draft, methodology; review & editing, All authors read and approved the final manuscript.

## Conflicts of interest

There are no conflicts to declare.

## Supplementary Material

RA-014-D4RA06529H-s001

RA-014-D4RA06529H-s002

RA-014-D4RA06529H-s003

RA-014-D4RA06529H-s004

RA-014-D4RA06529H-s005

RA-014-D4RA06529H-s006

RA-014-D4RA06529H-s007

RA-014-D4RA06529H-s008

RA-014-D4RA06529H-s009
