# Supplementary figures and images for: Four newly synthesized enones induce mitochondrial-mediated apoptosis and G2/M cell cycle arrest in colorectal and cervical cancer cells
Source: RSC Adv. 2024 Oct 25;14(46):33987–4004. doi: 10.1039/d4ra06529h (PMC11505670; doi:10.1039/d4ra06529h)

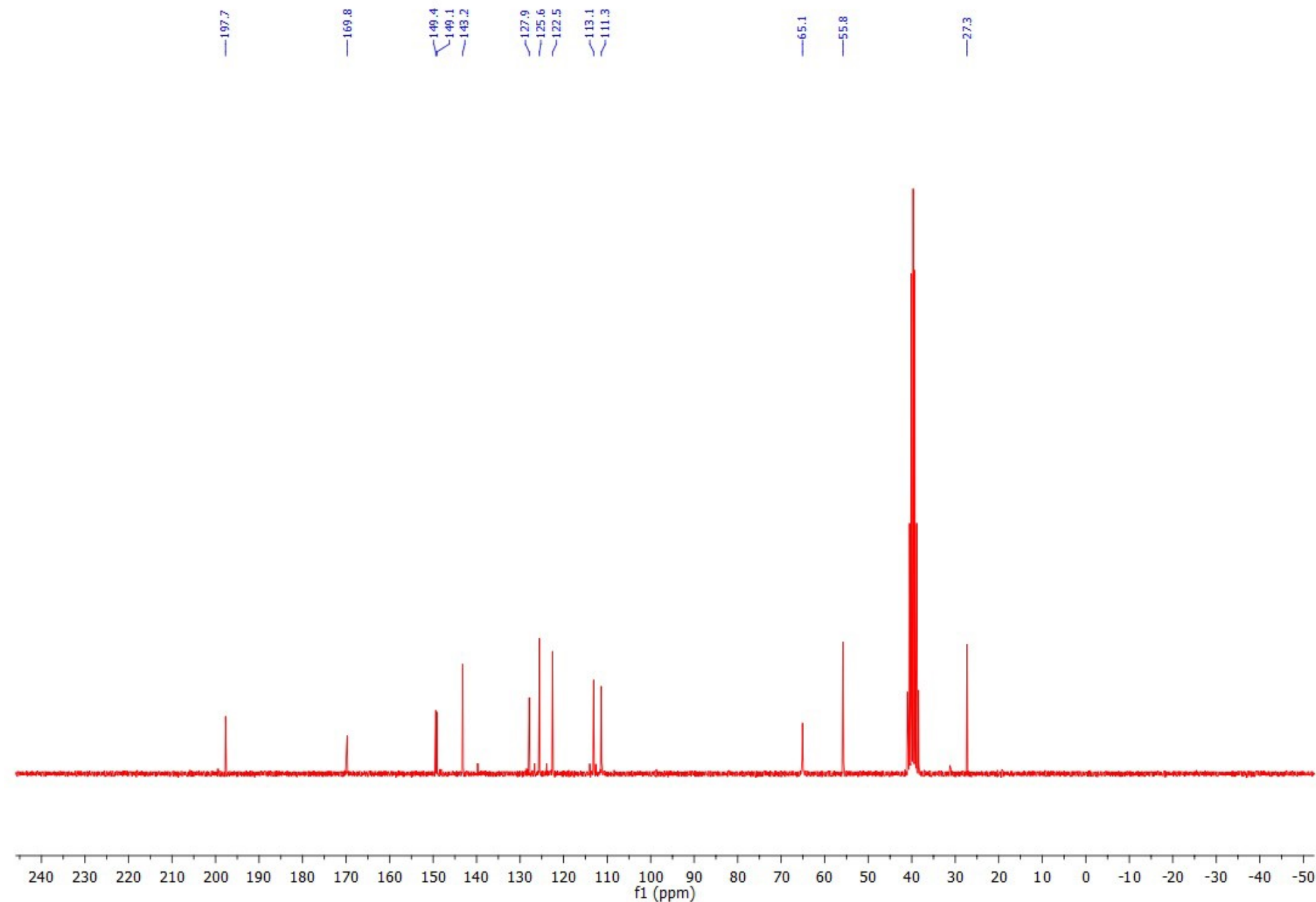

Supplement: RA-014-D4RA06529H-s001 [file RA-014-D4RA06529H-s001.pdf]

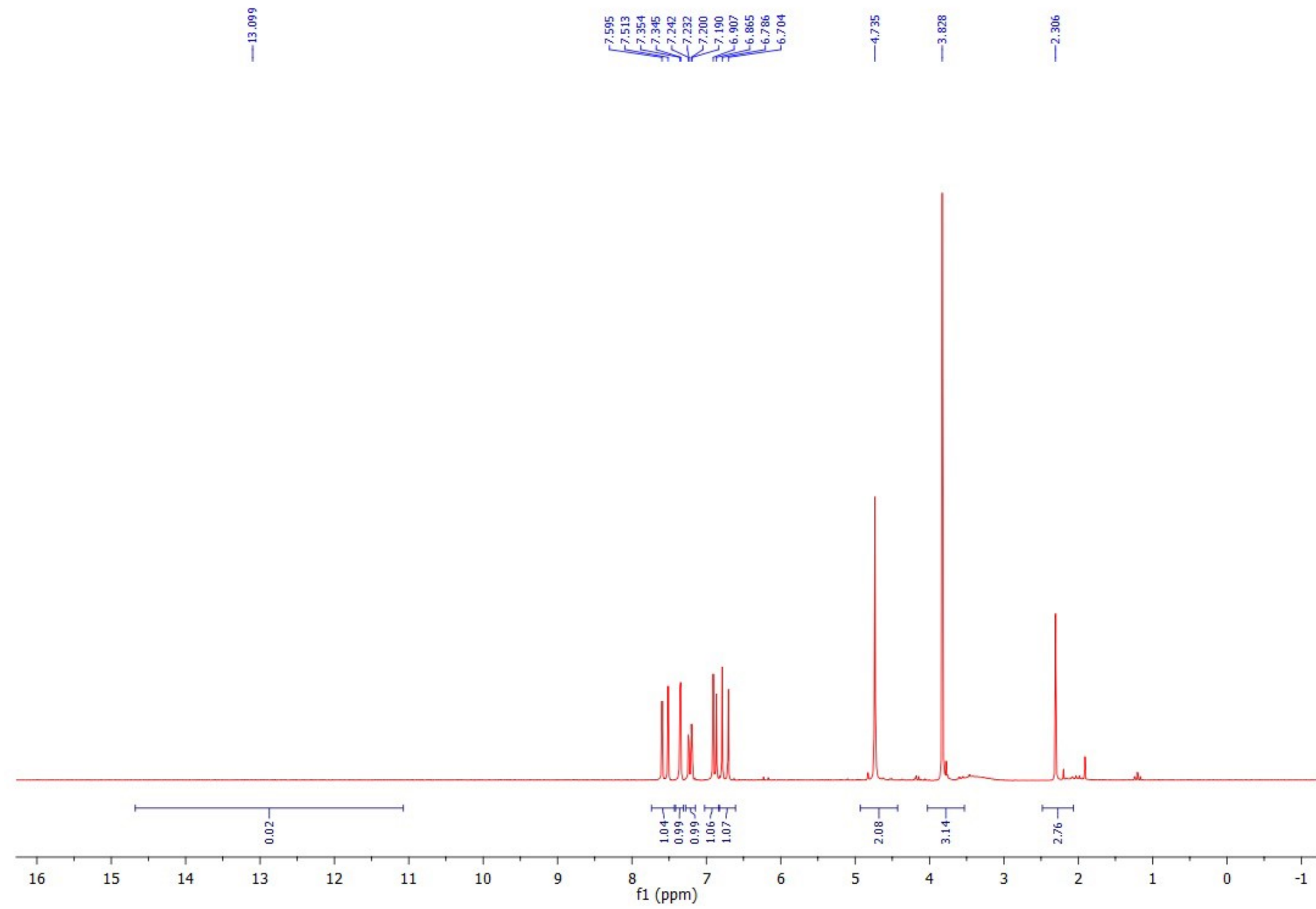

Supplement: RA-014-D4RA06529H-s002 [file RA-014-D4RA06529H-s002.pdf]

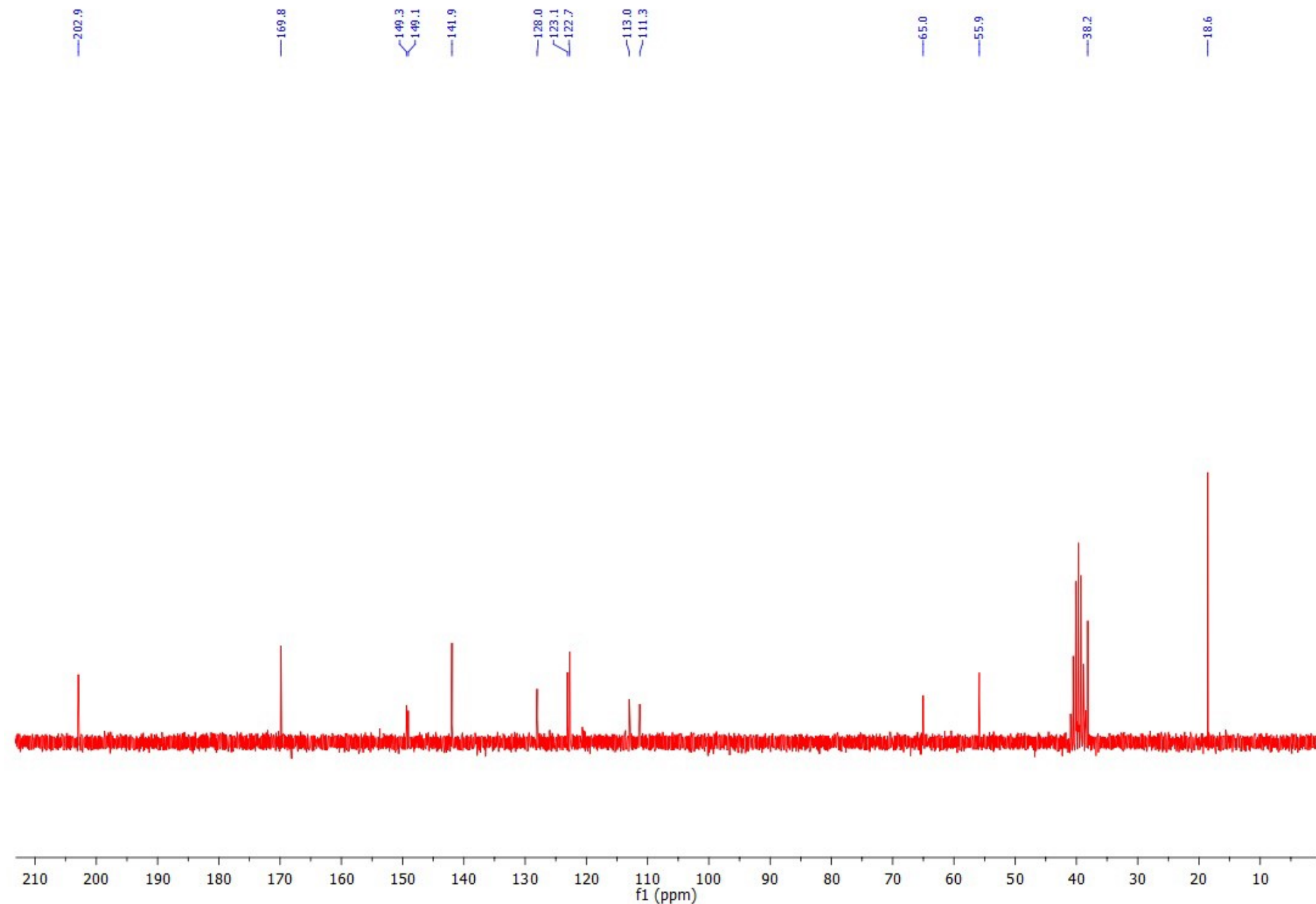

Supplement: RA-014-D4RA06529H-s003 [file RA-014-D4RA06529H-s003.pdf]

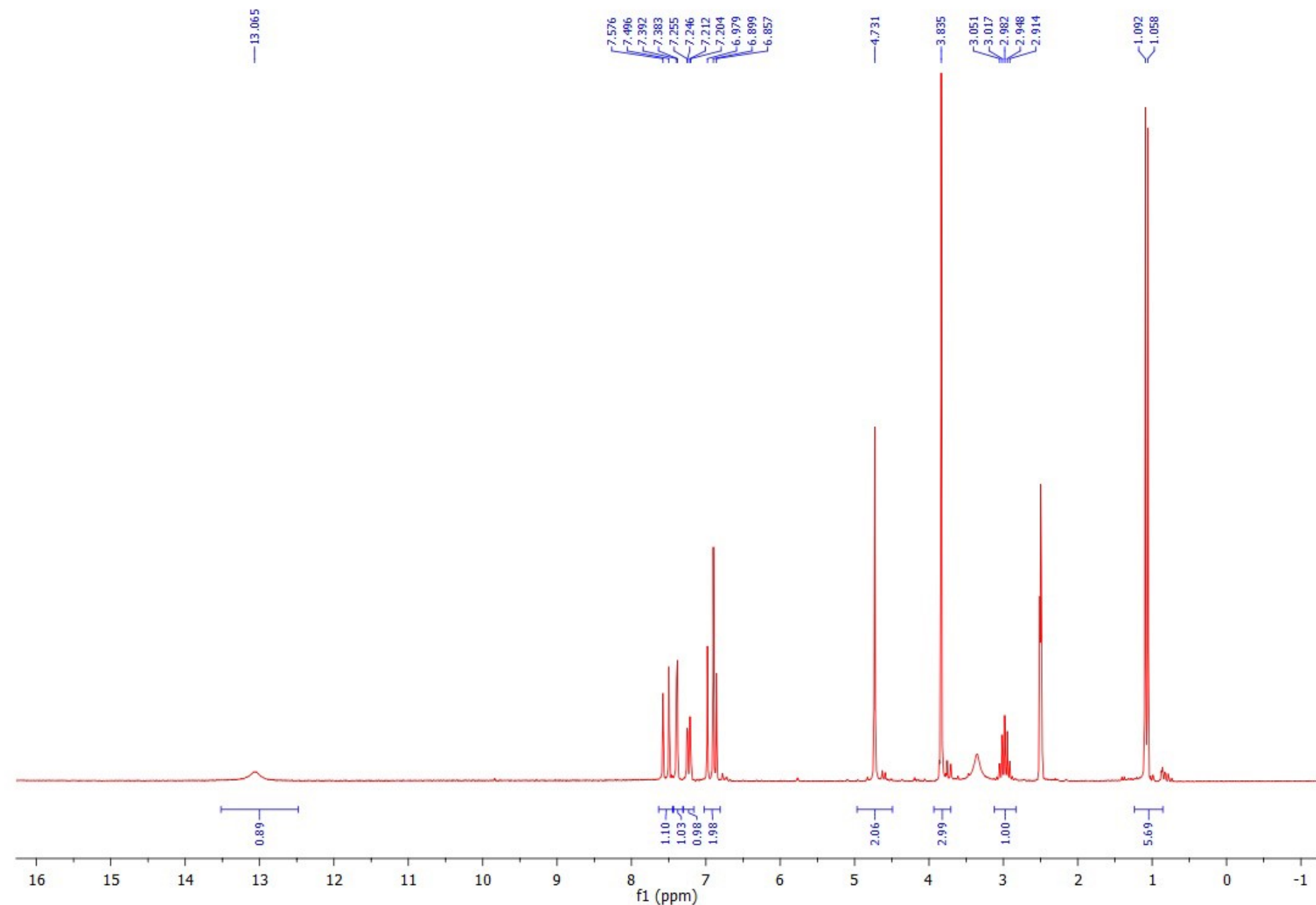

Supplement: RA-014-D4RA06529H-s004 [file RA-014-D4RA06529H-s004.pdf]

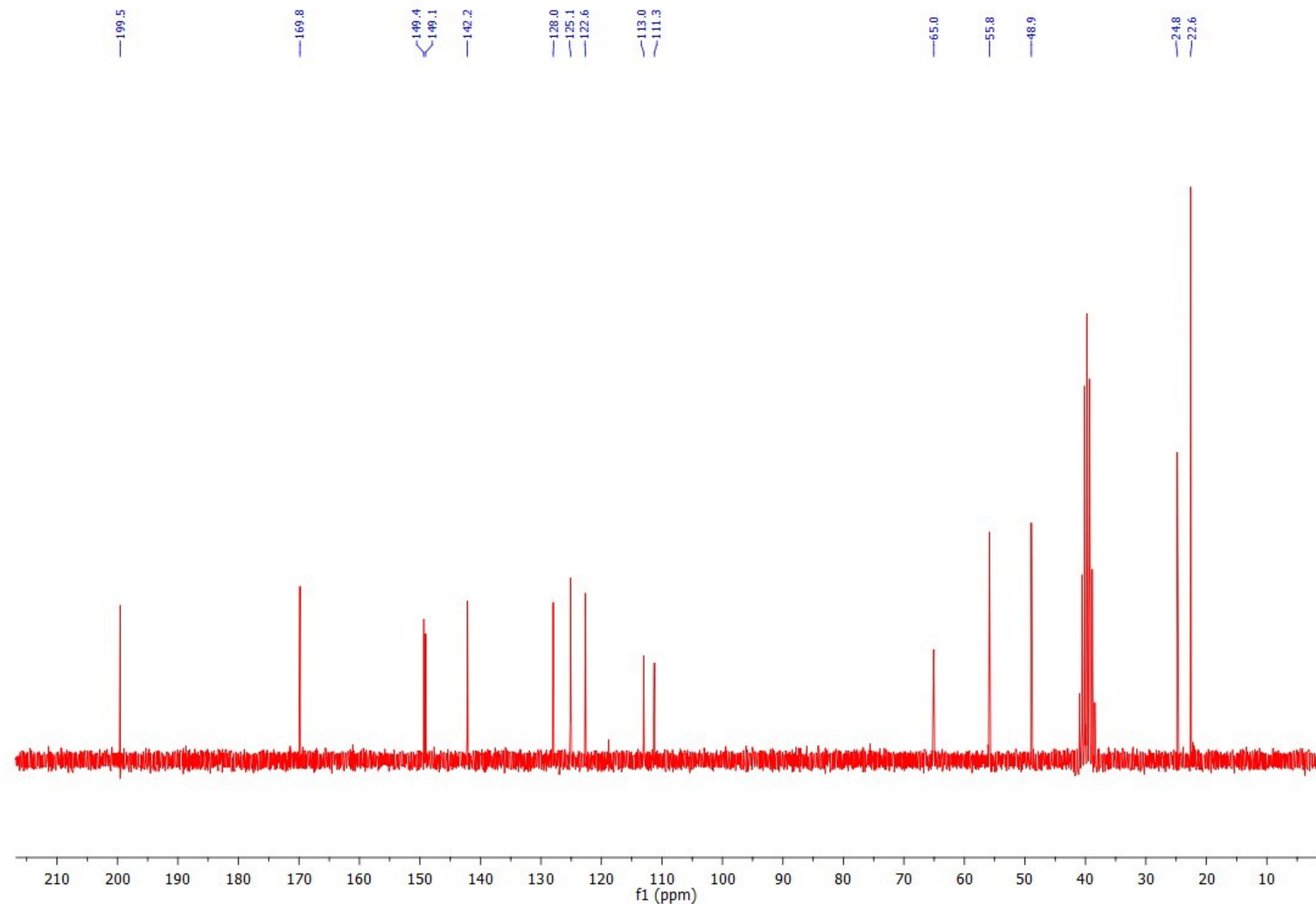

Supplement: RA-014-D4RA06529H-s005 [file RA-014-D4RA06529H-s005.pdf]

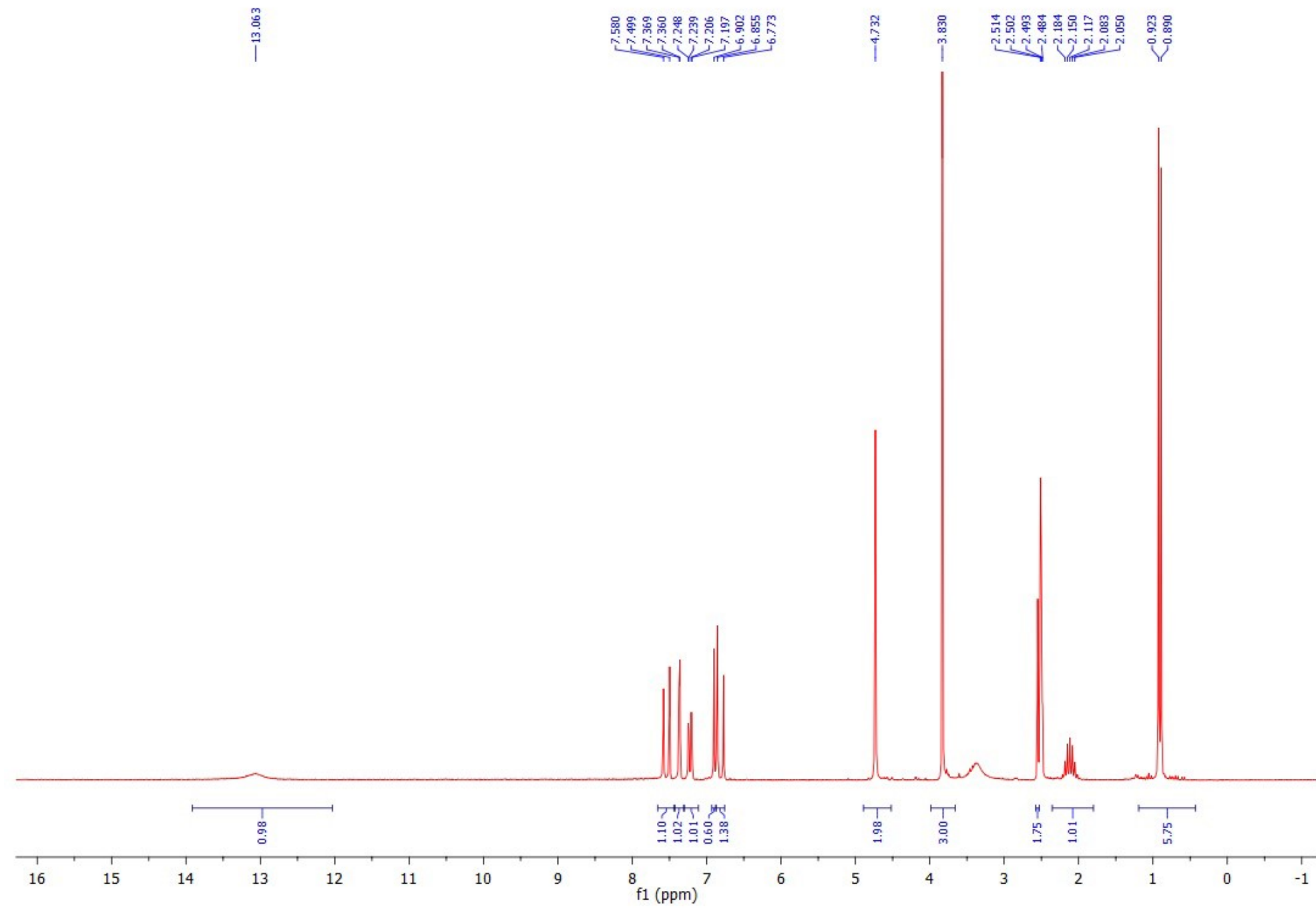

Supplement: RA-014-D4RA06529H-s006 [file RA-014-D4RA06529H-s006.pdf]

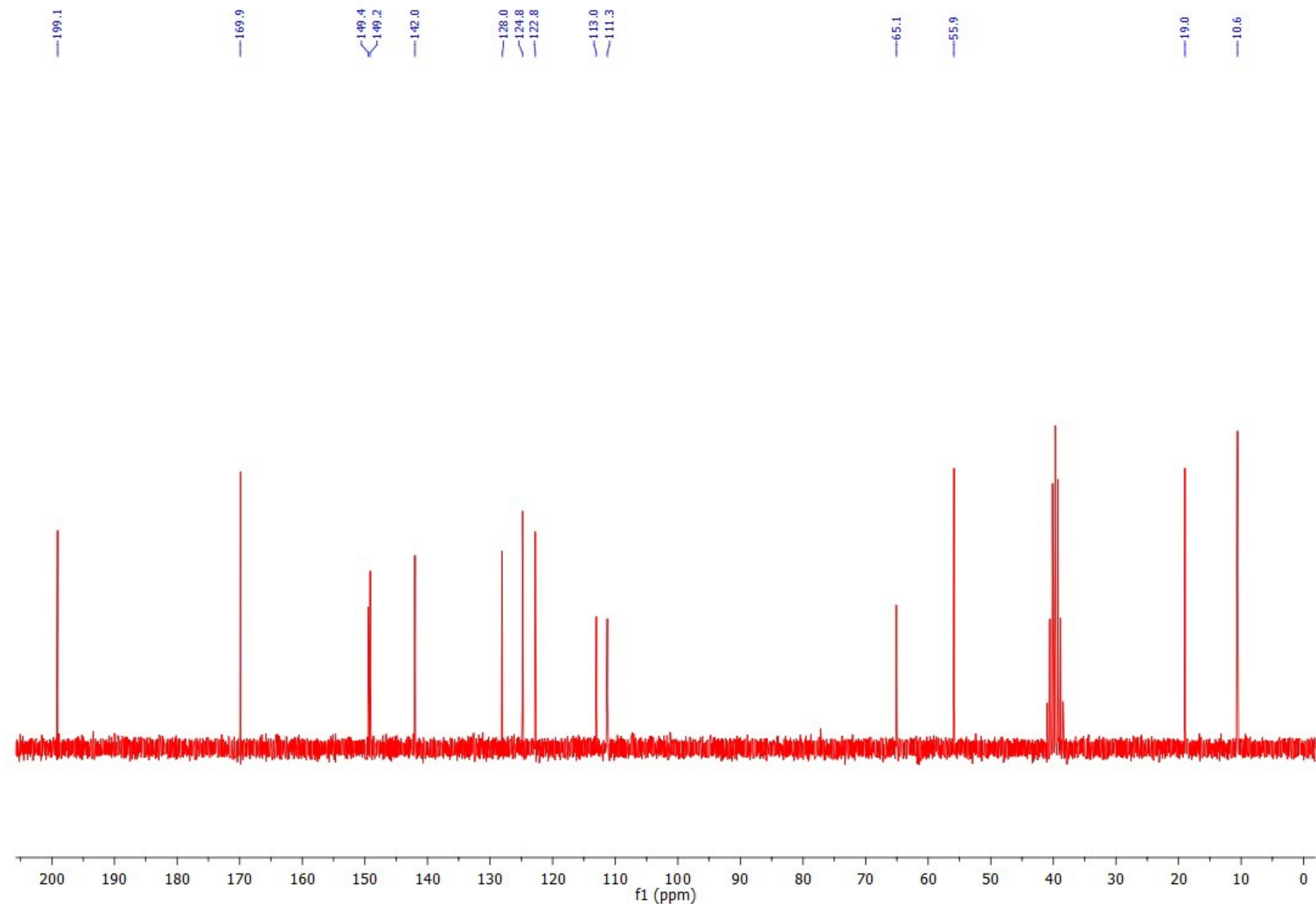

Supplement: RA-014-D4RA06529H-s007 [file RA-014-D4RA06529H-s007.pdf]

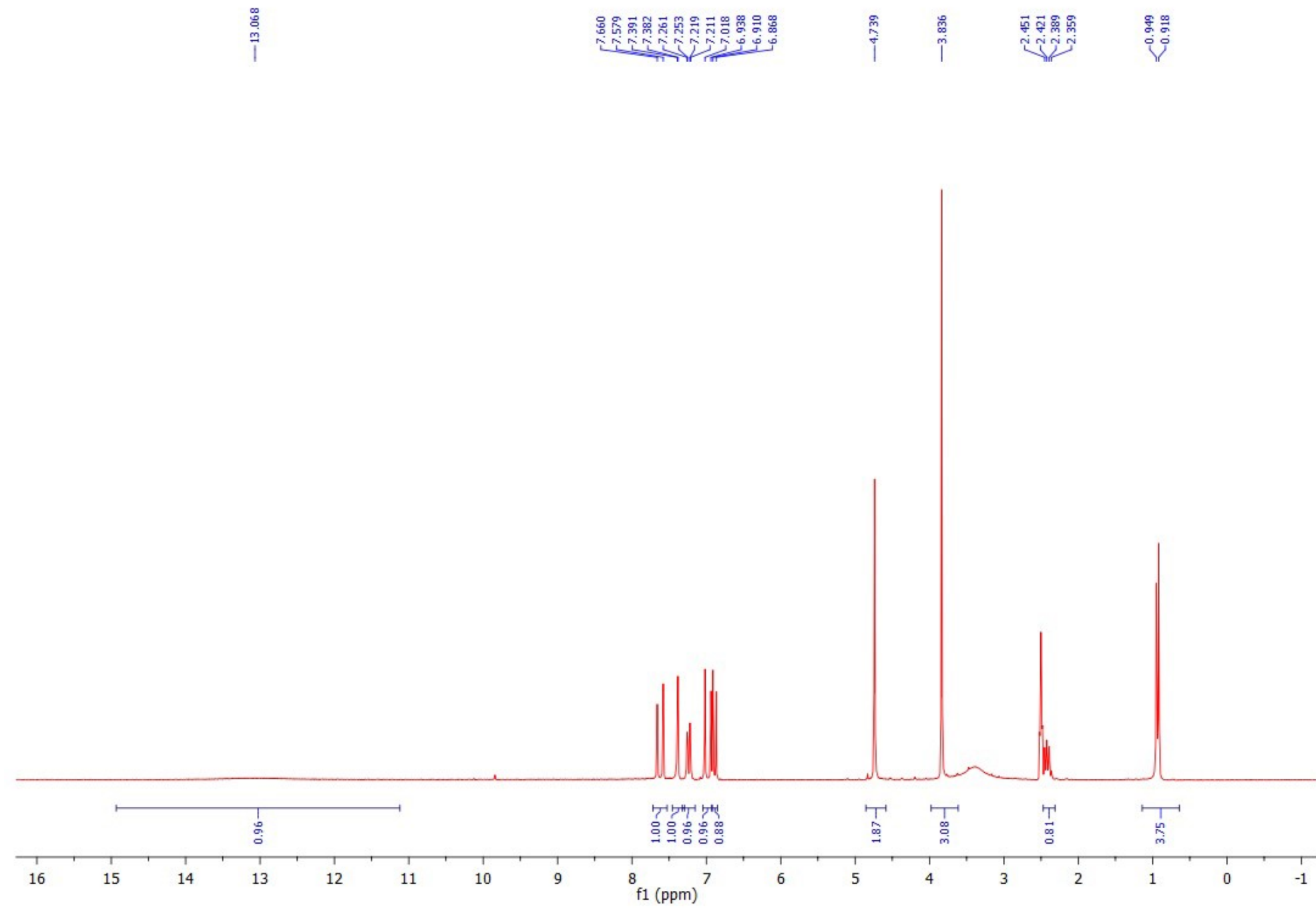

Supplement: RA-014-D4RA06529H-s008 [file RA-014-D4RA06529H-s008.pdf]
