# Supplementary material for: Four newly synthesized enones induce mitochondrial-mediated apoptosis and G2/M cell cycle arrest in colorectal and cervical cancer cells
Source: RSC Adv. 2024 Oct 25;14(46):33987–4004. doi: 10.1039/d4ra06529h (PMC11505670; doi:10.1039/d4ra06529h)
Supplement: RA-014-D4RA06529H-s009 [file RA-014-D4RA06529H-s009.pdf]

Supplementary Information (SI) for RSC Advances.
This journal is © The Royal Society of Chemistry 2024

RSC Advances

Electronic Supplementary Information associated with the paper

Four newly synthesized Enones induce mitochondrial-mediated apoptosis and G2/M cell cycle arrest in colorectal and cervical cancer cells.

Marija Bulić[a], Ivana Nikolić[b, c], Marina Mitrović[b], Jovana Muškinja[d], Tamara Todorović[d] and Marija Anđelković[b, c*]

✉ Marija Anđelković
marijabcd@gmail.com

[a]*University clinical Centre of Serbia, Centre of medical biochemistry. Belgrade, Serbia, Pasterova 2, 11000 Belgrade, Serbia*

[b]*University of Kragujevac, Serbia, Faculty of Medical Sciences, Department of Biochemistry, Svetozara Markovića 69, 34000 Kragujevac*

[c]*Centre for Research on Harmful Effects of Biological and Chemical Hazards, Kragujevac, Serbia*

[d]*University of Kragujevac, Institute for Information Technologies, Department of Science, Jovana Cvijica bb, 34000 Kragujevac, Serbia*

***(E)-2-(2-methoxy-4-(3-oxobut-1-en-1-yl)phenoxy)acetic acid*, E1**

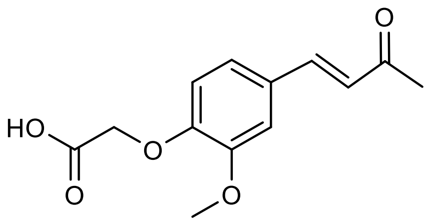

Yield: 85.2%; m.p. 143-144°C; IR (KBr): 3469, 2920, 1715, 1626, 1594, 1509, 1417, 1227, 1134, 1026, 972, 804 cm^{-1}; ^{1}HNMR (200 MHz, DMSO-d_6): 2.31 (*s*, 3H, CH_3CO), 3.83 (*s*, 3H, OCH_3), 4.74 (*s*, 2H, OCH_2), 6.74 (*d*, 1H, *J*=16.4 Hz, CH), 6.89 (*d*, 1H, *J*=8.4 Hz, Ar-H), 7.22 (*dd*, 1H, *J*=8.4, 2.0 Hz, Ar-H), 7.35 (*d*, 1H, *J*=1.6 Hz, Ar-H), 7.55 (*d*, 1H, *J*=16.4 Hz, CH), 13.10 (*br*. *s*, 1H, COOH); ^{13}CNMR (50 MHz, DMSO-d_6): 27.3, 55.8, 65.1, 111.3, 113.1, 122.5, 125.6, 127.9, 143.2, 149.1, 149.4, 169.8, 197.7 (CO).

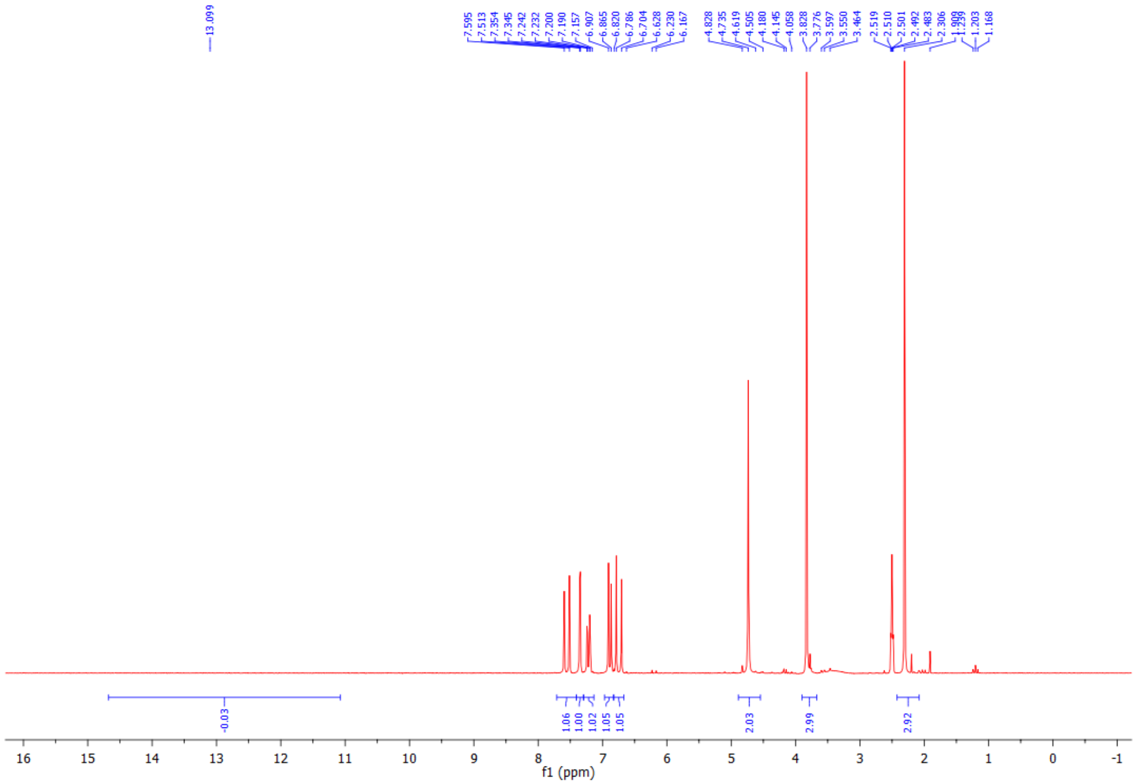

^{1}HNMR spectrum of compound **E1**

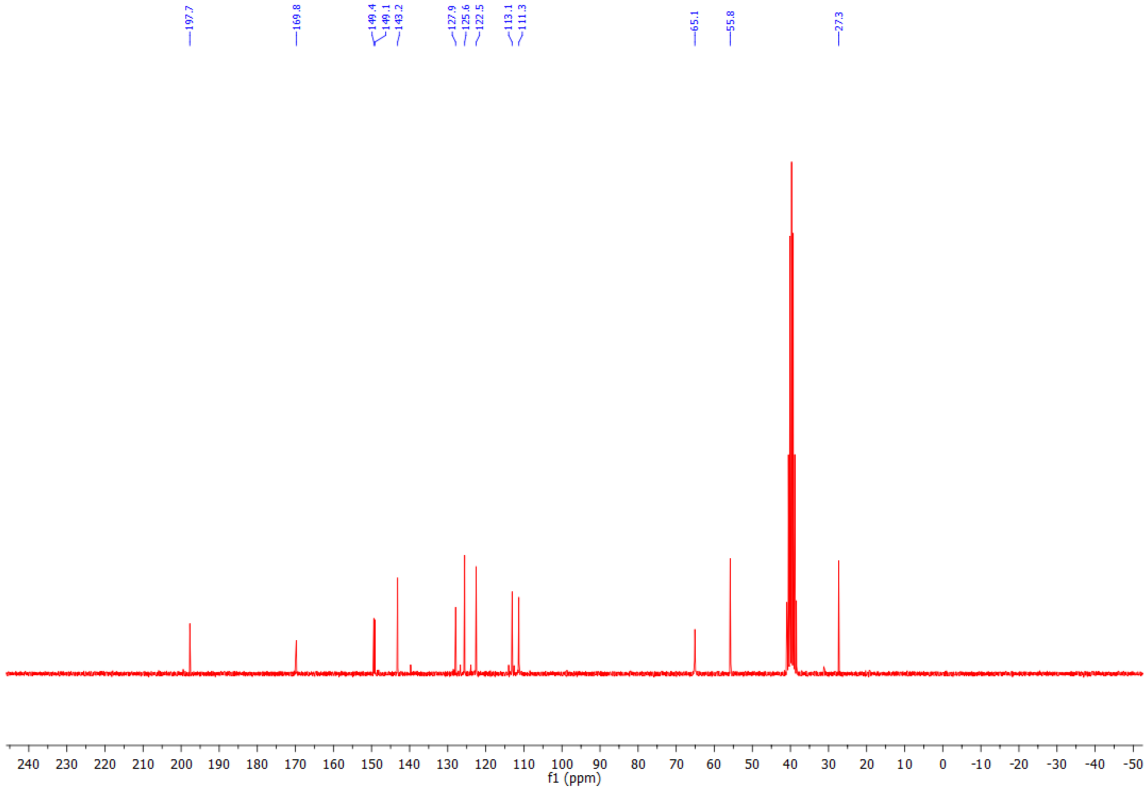

^{13}C NMR spectrum of compound **E1**

(E)-2-(2-methoxy-4-(4-methyl-3-oxopent-1-en-1-yl)phenoxy)acetic acid, **E2**

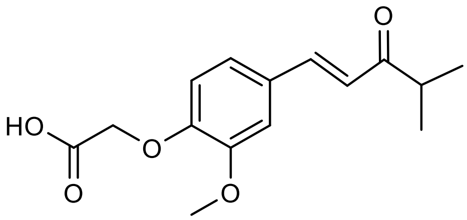

Yield: 62.7%; m.p. 115-116°C; IR (KBr): 3508, 2911, 1755, 1575, 1509, 1423, 1270, 1207, 1141, 1012, 977, 795 cm^{-1}; ^{1}HNMR (200 MHz, DMSO-d_6): 1.07 (*d*, 6H, *J*=6.8 Hz, $2CH_3$), 2.91-3.05 (*m*, 1H, C<u>H</u>$(CH_3)_2$), 3.83 (*s*, 3H, OCH_3), 4.73 (*s*, 2H, OCH_2), 6.86-6.98 (*m*, 1H, Ar-H), 6.94 (*d*, 1H, *J*=16.0 Hz, CH), 7.23 (*dd*, 1H, *J*=8.4, 1.8 Hz, Ar-H), 7.39 (*d*, 1H, *J*=1.8 Hz, Ar-H), 7.54 (*d*, 1H, *J*=16.0 Hz, CH), 13.09 (*br. s*, 1H, COOH); ^{13}CNMR (50 MHz, DMSO-d_6): 18.6, 38.2, 55.9, 65, 111.3, 113, 122.7, 123.1, 128, 141.9, 149.3, 149.4, 169.8, 202.9 (CO).

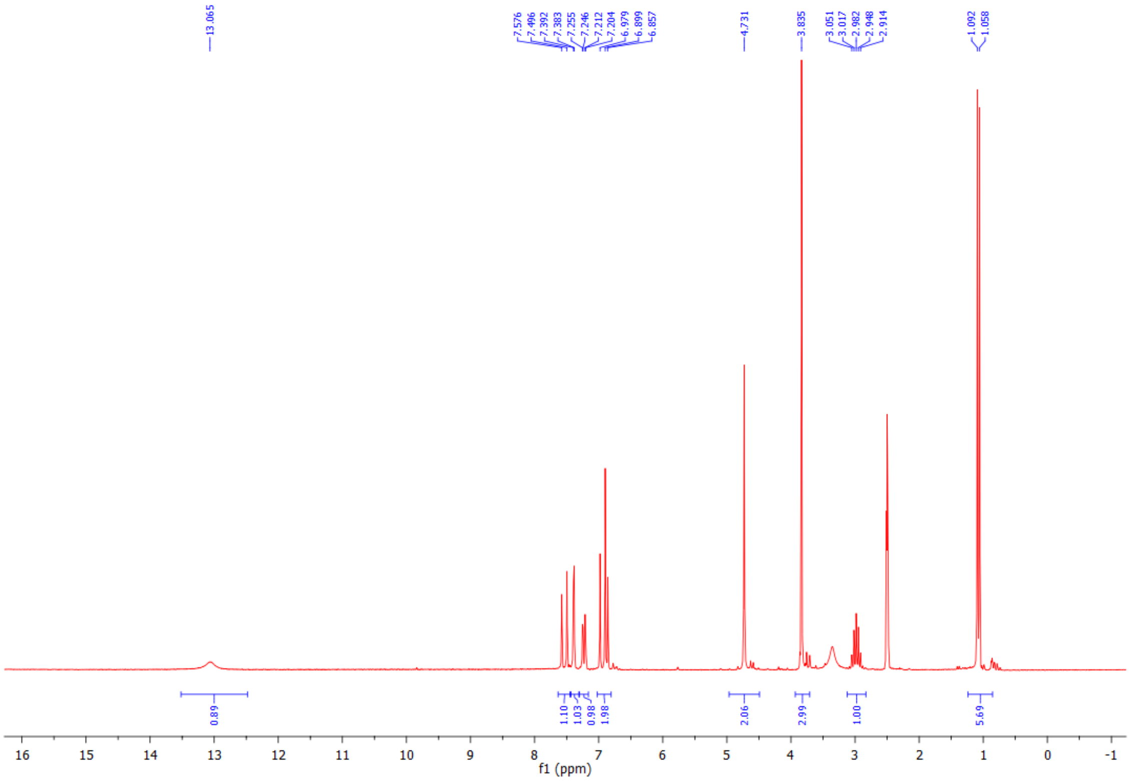

^{1}H NMR spectrum of compound **E2**

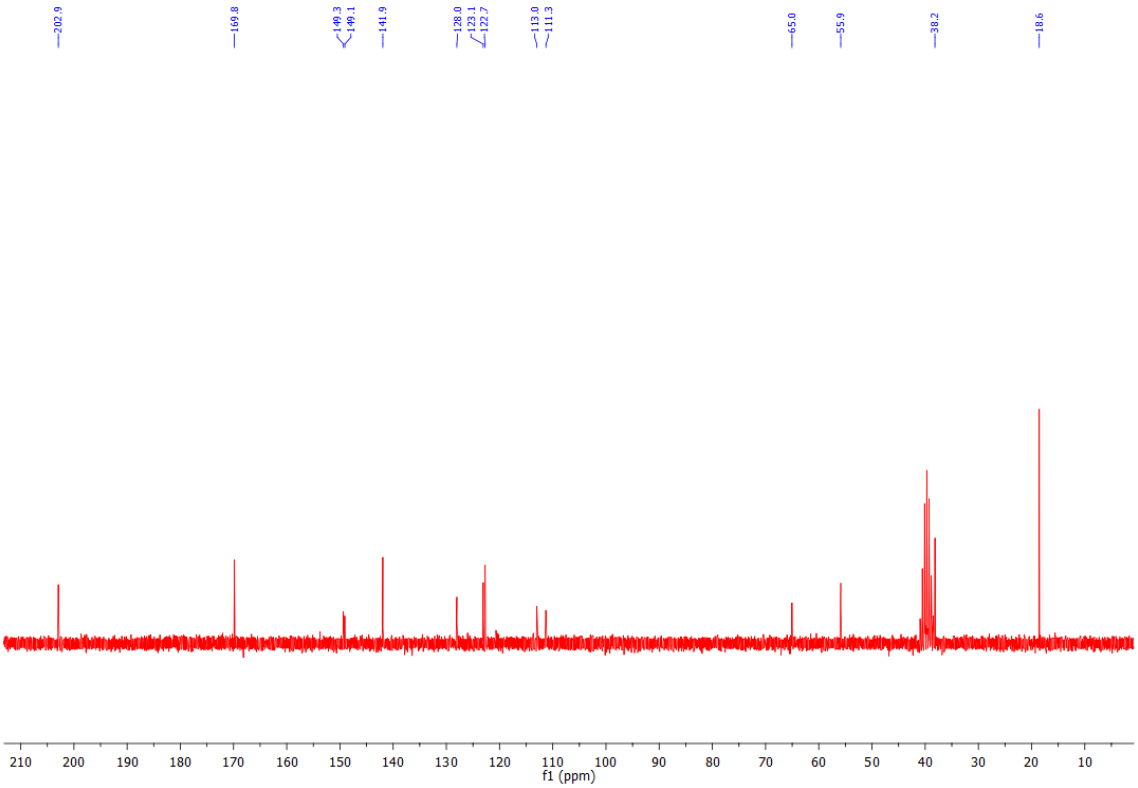

^{13}C NMR spectrum of compound **E2**

***(E)-2-(2-methoxy-4-(5-methyl-3-oxohex-1-en-1-yl)phenoxy)acetic acid*, E3**

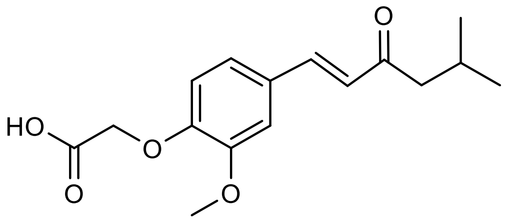

Yield: 93.2%; m.p. 117-118°C; IR (KBr): 3494, 2957, 1748, 1578, 1509, 1421, 1267, 1208, 1137, 1030, 819, 793 cm^{-1}; ^{1}HNMR (200 MHz, DMSO-d$_6$): 0.91 (*d*, 6H, *J*=6.6 Hz, 2CH_3), 2.05-2.18 (*m*, 1H, $C\underline{H}(CH_3)_2$), 2.53 (*d*, 2H, *J*=7.0 Hz, $C\underline{H_2}CH(CH_3)_2$), 3.83 (*s*, 3H, OCH_3), 4.73 (*s*, 2H, OCH_2), 6.81 (*d*, 1H, *J*=16.4 Hz, CH), 6.90 (*s*, 1H, Ar-H), 7.22 (*dd*, 1H, *J*=8.4, 1.8 Hz, Ar-H), 7.36 (*d*, 1H, *J*=1.8 Hz, Ar-H), 7.54 (*d*, 1H, *J*=16.2 Hz, CH), 13.06 (*br. s*, 1H, COOH); ^{13}CNMR (50 MHz, DMSO-d$_6$): 22.6, 24.8, 48.9, 55.8, 65, 111.3, 113, 122.6, 125.1, 128, 142.2, 149.1, 149.4, 169.8, 199.5 (CO).

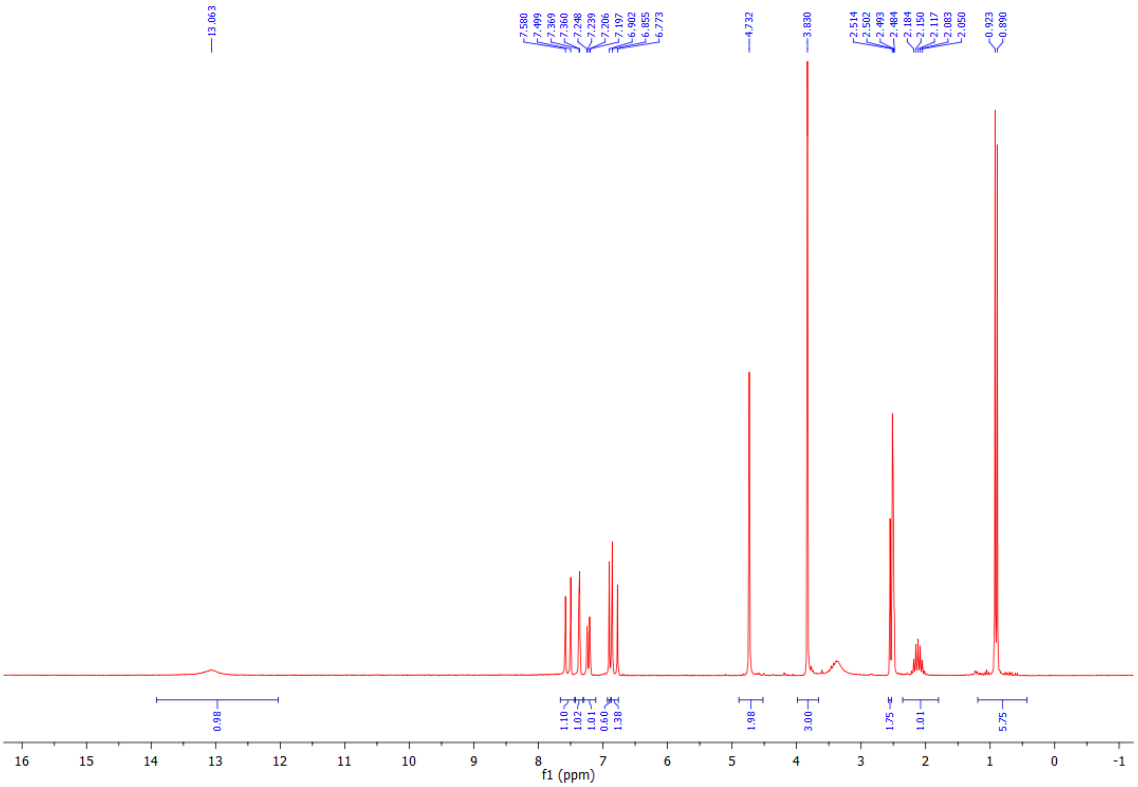

^{1}H NMR spectrum of compound **E3**

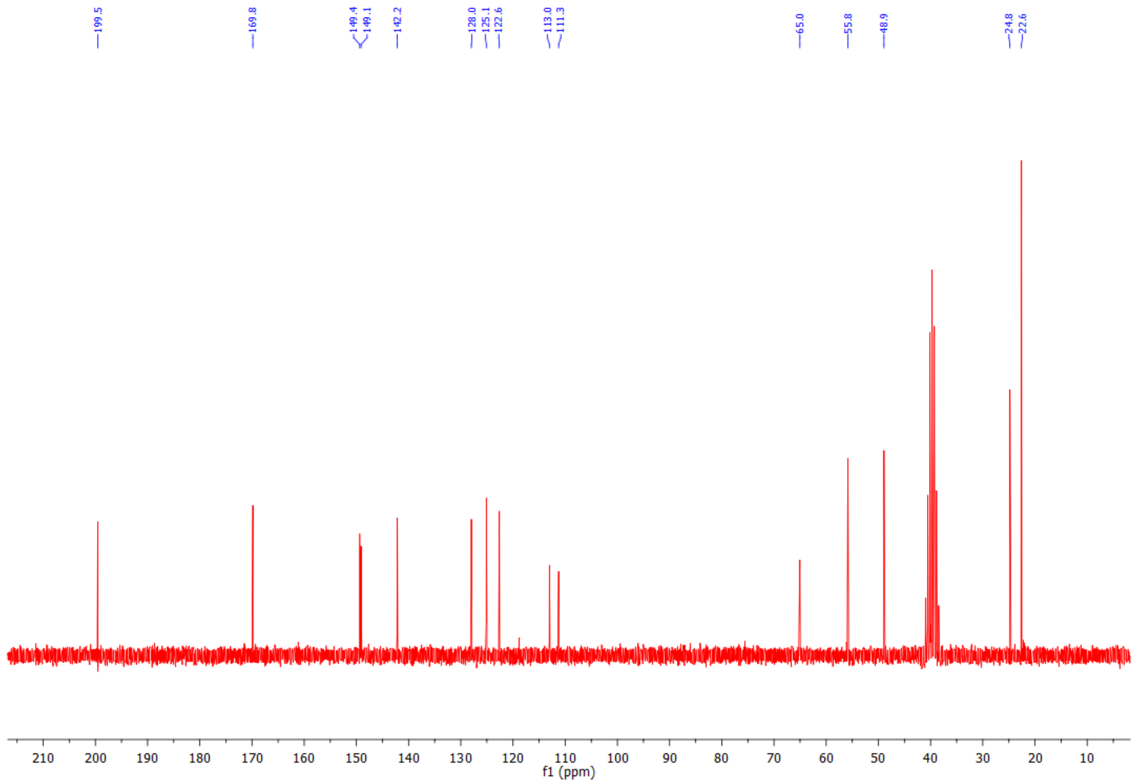

^{13}C NMR spectrum of compound **E3**

(E)-2-(4-(3-cyclopropyl-3-oxoprop-1-en-1-yl)-2-methoxyphenoxy)acetic acid, **E4**

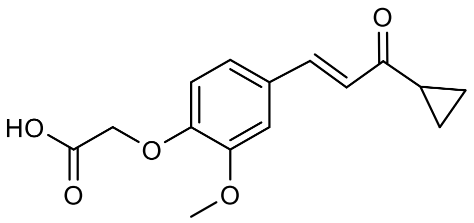

Yield: 90.8%; m.p. 89-90°C; IR (KBr): 3416, 1722, 1633, 1510, 1426, 1393, 1224, 1144, 1077, 989, 787 cm^{-1}; ^{1}HNMR (200 MHz, DMSO-d_6): 0.93 (*d*, 4H, *J*=6.2 Hz, $2CH_2$), 2.36-2.45 (*m*, 1H, CH), 3.84 (*s*, 3H, OCH_3), 4.74 (*s*, 2H, OCH_2), 6.89 (*d*, 1H, *J*=8.4 Hz, Ar-H), 6.98 (*d*, 1H, *J*=16.0 Hz, CH), 7.24 (*dd*, 1H, *J*=8.4, 1.6 Hz, Ar-H), 7.39 (*d*, 1H, *J*=1.8 Hz, Ar-H), 7.62 (*d*, 1H, *J*=16.2 Hz, CH), 13.07 (*br. s*, 1H, COOH); ^{13}CNMR (50 MHz, DMSO-d_6): 10.6, 19, 55.9, 65.1, 111.3, 113, 122.8, 124.8, 128, 142, 149.2, 149.4, 169.9, 199.1 (CO).

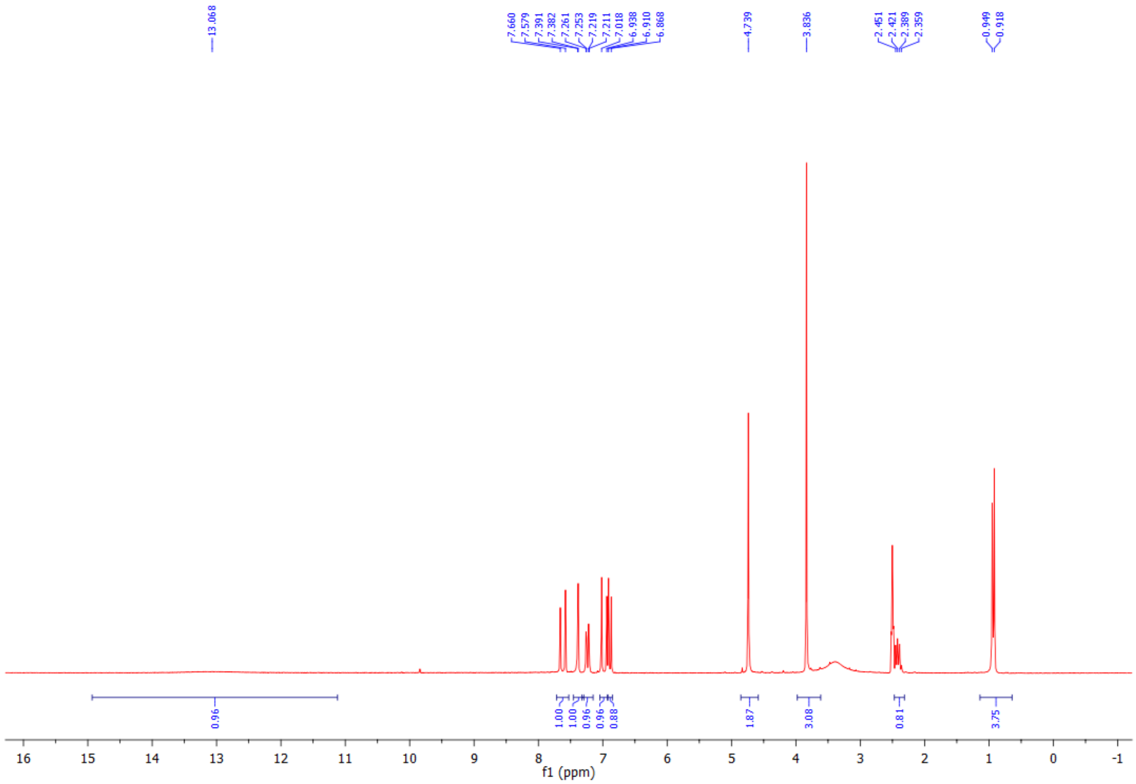

^{1}H NMR spectrum of compound **E4**

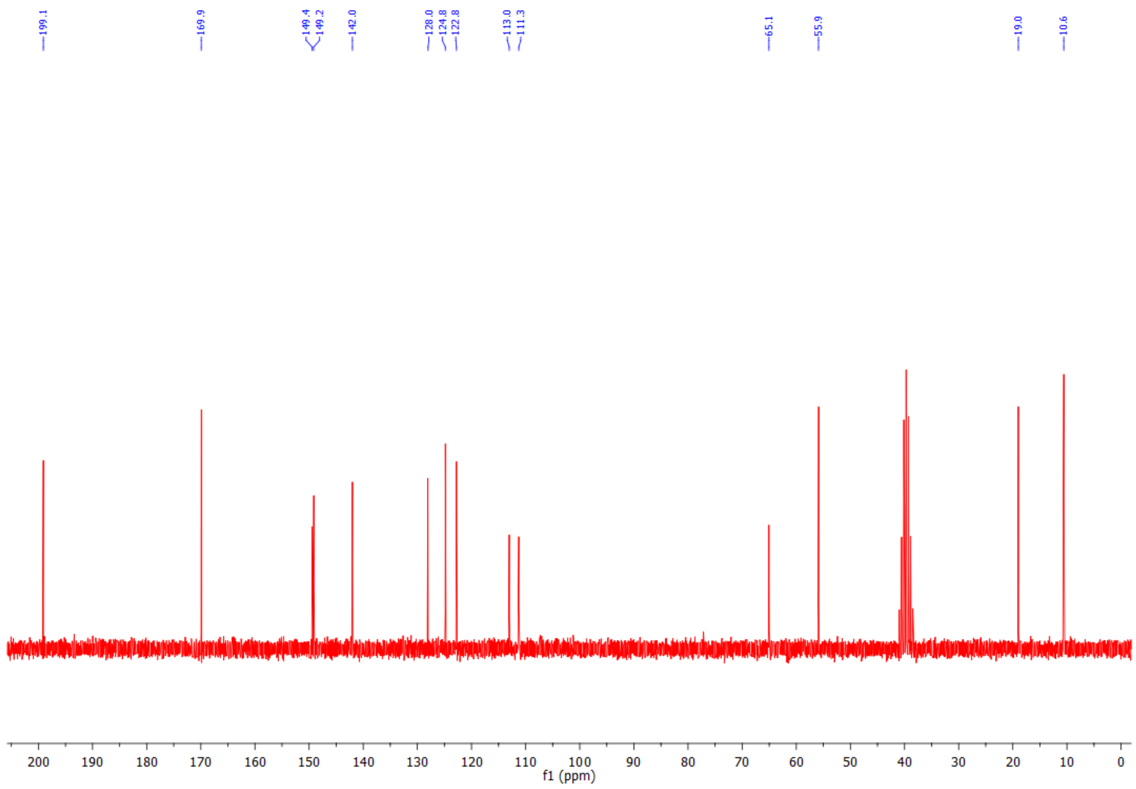

^{13}C NMR spectrum of compound **E4**
